# Prediction of Antibiotic Susceptibility Profiles of *Vibrio cholerae* Isolates From Whole Genome Illumina and Nanopore Sequencing Data: CholerAegon

**DOI:** 10.3389/fmicb.2022.909692

**Published:** 2022-06-22

**Authors:** Valeria Fuesslin, Sebastian Krautwurst, Akash Srivastava, Doris Winter, Britta Liedigk, Thorsten Thye, Silvia Herrera-León, Shirlee Wohl, Jürgen May, Julius N. Fobil, Daniel Eibach, Manja Marz, Kathrin Schuldt

**Affiliations:** ^1^Infectious Disease Epidemiology Department, Bernhard Nocht Institute for Tropical Medicine, Hamburg, Germany; ^2^RNA Bioinformatics and High-Throughput Analysis, Friedrich Schiller University Jena, Jena, Germany; ^3^German Center for Infection Research (DZIF), Hamburg-Lübeck-Borstel-Riems, Hamburg, Germany; ^4^National Center of Microbiology, Institute of Health Carlos III, Madrid, Spain; ^5^Department of Epidemiology, Johns Hopkins Bloomberg School of Public Health, Baltimore, MD, United States; ^6^Tropical Medicine II, University Medical Center Hamburg-Eppendorf (UKE), Hamburg, Germany; ^7^Department of Biological, Environmental and Occupational Health Sciences, School of Public Health, University of Ghana, Accra, Ghana

**Keywords:** antimicrobial resistance, AMR, *Vibrio cholerae*, nanopore, MinION, Illumina

## Abstract

During the last decades, antimicrobial resistance (AMR) has become a global public health concern. Nowadays multi-drug resistance is commonly observed in strains of *Vibrio cholerae*, the etiological agent of cholera. In order to limit the spread of pathogenic drug-resistant bacteria and to maintain treatment options the analysis of clinical samples and their AMR profiles are essential. Particularly, in low-resource settings a timely analysis of AMR profiles is often impaired due to lengthy culturing procedures for antibiotic susceptibility testing or lack of laboratory capacity. In this study, we explore the applicability of whole genome sequencing for the prediction of AMR profiles of *V. cholerae*. We developed the pipeline CholerAegon for the *in silico* prediction of AMR profiles of 82 *V. cholerae* genomes assembled from long and short sequencing reads. By correlating the predicted profiles with results from phenotypic antibiotic susceptibility testing we show that the prediction can replace *in vitro* susceptibility testing for five of seven antibiotics. Because of the relatively low costs, possibility for real-time data analyses, and portability, the Oxford Nanopore Technologies MinION sequencing platform—especially in light of an upcoming less error-prone technology for the platform—appears to be well suited for pathogen genomic analyses such as the one described here. Together with CholerAegon, it can leverage pathogen genomics to improve disease surveillance and to control further spread of antimicrobial resistance.

## 1. Introduction

The vast number of pathogenic bacterial strains that are resistant to antimicrobial agents today has become a difficult public health issue (World Health Organization, 2014). Early identification and epidemiological surveillance of resistance in bacteria are key in order to guide the appropriate use of antimicrobials and to prevent further spread of antimicrobial resistance (AMR).

Commonly, antimicrobial susceptibility testing (AST) is performed phenotypically *in vitro* by a microbiological procedure based on the growth of a single bacterial isolate in the presence or absence of an anti-microbiological agent. Especially for clinical settings phenotypic AST represents an important method which can deliver robust and reproducible results. However, the phenotypic AST is limited in the sense that it only allows for assessing resistance to known antibiotics and due to the time needed for the growth of the microorganism results are often not provided timely. Depending on the microorganism in question it may take up to several days after the isolation of a clinical isolate until an AMR profile can be provided (Didelot et al., 2012; van Belkum et al., 2020). Moreover, *in vitro* AST is difficult to scale-up and requires laboratory facilities with suitable equipment, trained microbiologists, as well as the availability of the antibiotics in question. In low- and middle-income countries (LMICs), where AMR rates appear to be higher than in high income countries (HICs) (World Health Organization, 2021), these requirements are often not given and the lack of laboratory capacity hinders the necessary assessment of AMR in bacteria causing life-threatening infections.

AMR in bacteria is genetically encoded and can be intrinsically mediated or acquired by *de novo* mutations or the intake of new resistance genes from other organisms. Hence, an alternative approach to analyze the presence of AMR in bacteria is whole genome analysis, allowing to quickly identify the presence of resistance genes and resistance-conferring mutations (Didelot et al., 2012; Boolchandani et al., 2019). For instance, whole genome sequencing methods have been successfully implemented to accelerate diagnostics and prediction of drug resistance profiles of *Mycobacterium tuberculosis* (Consortium et al., 2018; Doyle et al., 2018). Multiple methods have been developed to detect AMR genes directly from sequencing read from isolates or from metagenomic reads, as well as from assembled genomes (for a detailed review see Boolchandani et al., 2019). Most tools utilize alignment and/or HMM strategies to find genes with individual curated AMR gene reference databases, e.g., Resistance Gene Identifier (CARD) (Alcock et al., 2019), Resfinder (Bortolaia et al., 2020), ARG-ANNOT (Gupta et al., 2014), or the NCBI database used for AMRfinderPlus (Feldgarden et al., 2021). Abricate (Seemann, 2016) integrates all these databases with a BLAST alignment method, but is restricted to homolog models.

Both, traditional short read sequencing (e.g., Illumina) and long read sequencing (e.g., Oxford Nanopore Technologies - ONT, Pacific Biosciences) are commonly used for the genome assembly of bacterial isolates (De Maio et al., 2019). Long reads enable higher contiguity and can resolve long repeat regions, while short reads have lower error rates. Hybrid approaches harness the advantages of both types, leading to complete genomes with few assembly errors (De Maio et al., 2019). The portability of the MinION device by ONT allows in-field sequencing and rapid result turnaround, suited for applications using direct patient samples (Maestri et al., 2019; Leggett et al., 2020).

Cholera is an acute diarrheal disease and nowadays considered a neglected disease mainly affecting populations in low-income countries, where contaminated water and poor sanitation are the main drives for the bacterial spread (Lippi et al., 2016). With approximately 2.9 million cases and an estimated 95,000 cholera-associated deaths each year the vast majority of cholera cases are observed on the African continent (Clemens et al., 2017). The disease's causing agent is the Gram-negative bacterium *Vibrio cholerae*, which belongs to the family of *Vibrionaceae*, mostly found in aquatic environments (Momba and El-Liethy, 2017). *V. cholerae* comprises more than 200 O serogroups, of which serogroups O1 and O139 strains are pathogenic for the human host (Shimada et al., 1994; Momba and El-Liethy, 2017). The O1 serogroup is subdivided into two biotypes, the classical and the El Tor biotype, the latter being responsible for the current pandemic (Ramamurthy et al., 2019).

The standard treatment for acute watery diarrhoea caused by *V. cholerae* is oral rehydration therapy (ORT) (Nalin and Cash, 2018). Antibiotic therapy is recommended for severely ill cholera patients, who have severe dehydration and continue to pass a large volume of stool during rehydration treatment, pregnant women, and patients with comorbidities, such as an infection with the human immunodeficiency virus (HIV) or severe malnutrition. The administration of antibiotic regimen helps to shorten the length of disease and to limit the number of infectious bacteria in the stool (Ramamurthy et al., 2019; Kaunitz, 2020). In recent years, however, treatment failures due to the recurrent emergence of antimicrobial resistant *V. cholerae* have been observed frequently (Clemens et al., 2017). The occurrence of *V. cholerae* strains that are not susceptible to antibiotics is especially challenging since the bacteria are able to pass on resistance-mediating genetic sequences as part of a highly mobile elements conferring antimicrobial resistance to other bacterial species *via* horizontal gene transfer (Kitaoka et al., 2011; Verma et al., 2019; Das et al., 2020).

In this study, our overarching aim is to assess the applicability of whole genome sequencing with short and long reads for the prediction of clinically relevant AMR in *V. cholerae*. We subjected 82 *V. cholerae* isolates to whole genome sequencing and established a bioinformatic analysis workflow for the identification of genetic resistance loci in *V. cholerae*. Subsequently, we correlated the results from the whole genome analysis to results from phenotypic *in vitro* characterization by AST for the same isolates. Furthermore, we estimate time and costs needed to apply this approach for the prediction of AMR profiles from bacterial genomes.

## 2. Materials and Methods

### 2.1. *V. cholerae* Isolates

Patient samples were collected during cholera outbreaks in Ghana during the years 2011, 2012, and 2014 by the Ghanaian National Public Health & Reference Laboratory (NPHRL) as described by Eibach et al. (2016). In this study, 82 of the previously characterized *V. cholerae* isolates were subjected to whole genome sequencing (2011 *n* = 16, 2012 *n* = 8, 2014 *n* = 58).

### 2.2. Phenotypic Antibiotic Susceptibility Testing

The characterization of the phenotypic AST of the here sequenced isolates has been described previously (Eibach et al., 2016). Briefly, AST was performed for 80 isolates by the Kirby-Bauer disk diffusion method for the following seven antimicrobial drugs: ampicillin, chloramphenicol, gentamicin, nalidixic acid, sulfamethoxazole/trimethoprim, tetracycline and ciprofloxacin. Classifications of isolates into susceptible (S), susceptible dose-dependent (SD), intermediate (I), and resistant (R) phenotypes were done according to the 2015 Clinical and Laboratory Standards Institute (CLSI) guidelines (http//www.clsi.org) and when no interpretive criteria were available for *V. cholerae*, breakpoints for *Enterobacteriaceae* based on the 2015 European Committee on Antimicrobial Susceptibility Testing (EUCAST, http://www.eucast.org) were applied.

### 2.3. Culture and DNA Isolation

*Vibrio cholerae* isolates were stored in skim milk at -20°C. Culturing was performed on Columbia Agar with sheep blood. Plated bacteria were incubated at 26,5°C for 20–24 h and were then stored at 4°C for a maximum of 7 days. The LGC MasterPure Complete DNA and RNA Purification Kit (Lucigen Corporation, Cat. No. MC85200) was used for DNA extraction from the 82 *V. cholerae* isolates. Quality control of the isolated DNA was done by fluorometric quantification using the Quant-iT^*TM*^ broad-range dsDNA assay kit (Thermo Fisher Scientific) and gel electrophoresis.

### 2.4. Illumina Library Preparation and Sequencing

Illumina library preparation was performed using the Nextera XT Library Prep Kit and the IDT for Illumina Nextera DNA Unique Dual Indexes Set A (both Illumina Inc.). Sequencing was performed on Illumina NextSeq with the NextSeq 500/550 Mid Output Reagent Cartridge v2.5 (Illumina Inc). Quality assessments of short reads were performed using fastqc (Andrews, 2010). The number of reads etc. can be found in the results and Supplementary Material.

### 2.5. Oxford Nanopore Technologies Library Preparation and Sequencing

An input mass of 1 μg of DNA was used for each sample for library preparation, following the instructions of ONT 1D Native barcoding genomic DNA protocol from May 2018. The following procedures complemented the ONT protocol: DNA repair was carried out using the NEBNext Companion Module (New England Biolabs). This step was followed by the barcode ligation using NEB Blunt/TA Ligase (New England Biolabs) and EXP-NBD104 native barcode kit (ONT). After barcoding, 10-12 DNA samples were pooled resulting in a total of 1 μg DNA. For adapter ligation, the NEBNext Quick Ligation Module (New England Biolabs) and SQK-LSK109 ligation sequencing kit (ONT) were used. Throughout the protocol, AMPure XP beads (Beckman Coulter) were used to purify the samples. After DNA repair and barcode ligation the Elution Buffer (Promega Corporation) was selected for DNA Elution. Following adapter ligation, the DNA was eluted with elution buffer of SQK-LSK109 ligation sequencing kit (ONT). MinION sequencing was performed on the SpotON Flow Cell with the Flow Cell Priming Kit (Flow Cell Type R9.4.1, ONT). On average, ~250 ng of library DNA were loaded onto the flow cell for one run.

### 2.6. Basecalling of ONT Data

Conversion from raw data to nucleotide sequences (“basecalling”) of the raw nanopore data was performed with Guppy (available from Oxford Nanopore community portal) v5.0.16 using the “superior accuracy” configuration (dna_r9.4.1_450bps_sup.cfg). Guppy was run on HPC cluster nodes equipped with double NVIDIA Tesla GPUs (P100 16 GB or V100 16 GB) with the following parameter settings: enabled barcode demultiplexing for the utilized barcoding kit (–barcode_kits EXP-NDB104), usage of both GPUs (–device “cuda:0 cuda:1”), enabled barcode trimming (–trim_barcodes), disabled filtering of reads by median accuracy (–disable_qscore_filtering), and disabled sending of telemetry data (–disable_pings). The resulting fastq files were merged into a single fastq for each barcode. Quality control for the long reads was performed with pycoQC (Leger and Leonardi, 2019) v2.5.0.3.

### 2.7. CholerAegon Pipeline

We implemented the main analysis steps for short and long read data in an automated pipeline called CholerAegon. The pipeline is implemented in Nextflow (Tommaso et al., 2017) and Python3, and carries out short read assembly, long read assembly, and/or hybrid assembly, followed by assembly polishing, and finally assessment of resistance gene presence. Based on the found genes, CholerAegon predicts the antibiotic resistances of the sequenced isolates based on the CARD ontology (Alcock et al., 2019). We applied CholerAegon to 82 sequenced isolates. The individual steps of the pipeline are described in the next sections.

### 2.8. Genome Assembly

*A short read only* assembly was performed with Spades (Bankevich et al., 2012) v3.15.2 with default parameters except for the number of concurrent computation threads (-t 12). *Long read only* assembly was performed by running Flye (Kolmogorov et al., 2019) v2.9 with parameters set to treat input data as uncorrected nanopore reads (–nano-raw), and use multiple concurrent threads (–threads). Draft assemblies were polished with Medaka (ONT, 2018) v1.4.4 with the model (r941_min_sup_g507) which corresponds to the Guppy model used during basecalling. *Hybrid assembly* was performed by applying Pilon (Walker et al., 2014) v1.23 to the long read assembly, which uses the short reads to polish the long read draft assembly. Parameters were set to allocate 8 GB of memory (-Xmx8g) and use multiple threads (–threads 12). This approach harnesses the benefits of both data types: long reads lead to long contiguous sequences (and commonly complete genomes for bacterial samples like *V. cholerae*), and the very low error rate of the short reads can correct localized assembly errors. Average nucleotide identity (ANI) to the reference sequence (*V. cholerae* O1 biovar El Tor str. N16961, accession NC_002505.1) was determined with FastANI (Jain, 2018) v1.32 using default parameters.

### 2.9. Assessment of Resistance Loci

For each bacterial isolate and each assembly type, we performed the assessment of resistance gene presence with Resistance Gene Identifier (Alcock et al., 2019) (RGI) and Abricate (Seemann, 2016). RGI uses the full CARD database, which includes protein homolog models as well as sequence variant models. Abricate can only use protein homolog models and was used with the respective sequences for resistance genes in the CARD (Alcock et al., 2019) database. CholerAegon uses the RGI results in order to include available protein variant models. For each detected resistance gene, CholerAegon reviews Abricate results to yield hits with higher reference gene coverage. Finally, CholerAegon will filter out false positives by applying coverage and identity cutoffs (minimum 80 and 80%, respectively) on the remaining hits.

### 2.10. Comparison With Phenotypic Characterization

The correlation of the predicted antibiotic resistance from the whole genome sequencing data with the results from the phenotypic AST was performed with custom Python scripts. Specifically, we predicted an isolate as resistant to an antibiotic, if any of the detected genes have a confers_resistance_to_antibiotic relation to that antibiotic in the CARD ontology. Additionally, if a gene has a confers_resistance_to_drug_class related to a class of antibiotics, we considered the isolate resistant to all members of that drug class. Resistance against multi-component drugs are only assumed if resistance were predicted against all components. For each drug in the AST panel, we then compared the phenotypic AST result to the *in silico* predicted resistance profile.

We consider a prediction correct if for the “susceptible” (S) phenotype, no resistance has been predicted (no relevant gene or gene variant present), or if for the “intermediate” (I), “susceptible dose-dependent” (SD) and “resistant” (R) statuses, the resistance was predicted (relevant gene or gene variant found). All other combinations are considered false predictions.

## 3. Results and Discussion

### 3.1. CholerAegon—A Fully Automated Nextflow Pipeline for Antibiotic Resistance Prediction

**Figure 1:**
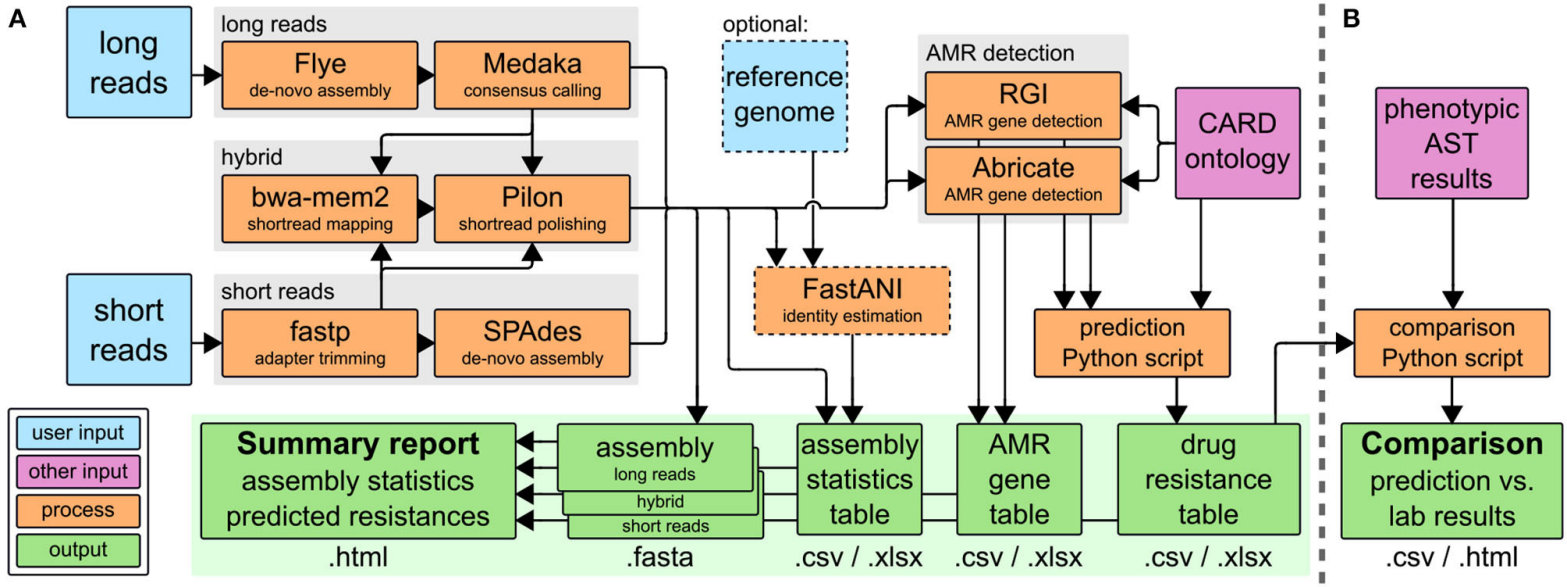
**(A)** The CholerAegon pipeline is implemented in Nextflow, Download at: https://github.com/RaverJay/CholerAegon. For each input sample, CholerAegon produces a genome assembly, basic assembly statistics, the predicted AMR genes, and subsequent drug resistance, and a summary report. **(B)** For this specific study of *Vibrio cholerae* isolates, AMR susceptibility test results were compared to *in silico* resistance predictions with a custom Python script. CARD, the comprehensive antibiotic resistance database (Alcock et al., 2019); AST, antimicrobial susceptibility testing.

The CholerAegon pipeline, refer to Figure 1A assembles reads, annotates antibiotic resistance genes based on homology models and predicts the resistance of any sequenced bacterial isolate. CholerAegon can process short reads such as Illumina reads, for which after an initial adapter trimming an assembly is created. In case long reads (created with e.g., ONT MinION) serve as input, a *de novo* assembly with a subsequent polishing step is given. In case long and shorts read are given only the hybrid assembly is used for further analysis.^1^ We evaluated our long-reads-first hybrid approach (Flye+Pilon) against Unicycler (Wick et al., 2017) v0.5.0, a short-reads-first hybrid assembler, and found that our approach outperformed Unicycler in terms of runtime and ANI (refer to Supplementary Figure 2). A basic assembly statistics table is given, which contains the total length of assemblies, number of contigs and N50 measure. In case a reference genome is given by –genome_reference, additionally, the ANI of the assembly is reported.

Furthermore, CholerAegon detects the AMR genes by combining the results of two tools: Abricate with RGI. While Abricate detects homologs more accurate than RGI, the latter is able to detect genetic variants of AMR genes. We chose these tools for their capability to detect more genes than other tools, such as AMRFinderPlus or Resfinder (see Supplementary Table 5). The default minimum thresholds for the identity and coverage of detected genes are set to 80% and 80%, respectively (adopted from Abricate), but can be customized with the parameters –min_identity_percent and –min_coverage_percent.

As a result, for each bacterial isolate, an antibiotic drug resistance profile is provided. Previously, two web-based tools have been developed that detect *V. cholerae* genotypes from sequencing data, which also allow to screen for the presence of AMR-conferring genes: CholeraeFinder 1.0 and VicPred (Siriphap et al., 2017; Lee et al., 2021). CholeraeFinder 1.0 uses Resfinder and VicPred RGI for AMR prediction. Due to the integration of the two best performing tools in our case, CholerAegon provides the most comprehensive AMR profiles (refer to Supplementary Table 5).

In case the user has performed wet-lab experimental AST, refer to Figure 1B, we give a comparison of predicted drug resistance (Figure 1A) with AST results.

Finally, the summary report (*.html output) collects all results as described above, complemented by the total run time.

The CholerAegon pipeline has been applied and in detail analyzed in this study for *V. cholerae*. However, individual processes of the pipeline can readily be improved or replaced. CholerAegon is configured to run all processes in virtual environments to ensure complete reproducibility. Docker containers are used for each program, which guarantees the exact same behavior independent of the hardware architecture used to run the pipeline. CholerAegon utilizes Bioconda's (Grüning et al., 2018) pre-built containers hosted on quay.io for most programs. Updating any process is easily accomplished by switching to a new container version. This modular design also allows for ease of expansion or adaptation for other bacterial pathogens.

### 3.2. *Vibrio cholerae* Assemblies Significantly Improved by Nanopore Sequencing

We obtained about 7 Mio. reads of length 73 nt (throughput 522 Mb and 111 X) per bacterial isolate with Illumina sequencing and on average 94,000 reads of about 5.8 kb in length (920 Mb throughput and 209 X) with ONT MinION sequencing. The length of long reads and subsequently the throughput varied by about one order of magnitude between the different bacterial isolates, refer to Supplementary Figure 1.

The advantage of the Illumina sequencing technology of a lower sequencing error rate is compromised by an average assembly length of about 4,042 Mb, whereas the long read sequencing strategy resulted in the full assembly length of 4,107 Mb, similar to a combined hybrid assembly strategy, refer to Table 1 and Supplementary Figure 1. Assemblies based on Nanopore data yielded the two chromosomes present in the *V. cholerae* genome *V. cholerae* (~3 Mb and ~1 Mb in length; Table 1) in complete contiguity for all isolates (erroneously fused in two strains). Illumina assemblies were highly fragmented but achieved a higher genome identity to the reference sequence. The hybrid assemblies harness the advantages of both sequencing approaches yielding few assembly errors at high sequence contiguity.

**Table 1:** General statistics of whole genome sequencing data.

	**Illumina**	**ONT**	**Hybrid**
# reads	7,055,057	93,778	–
Read length	74	5,768	–
Throughput	521,8 Mb	920,0 Mb	–
Coverage	111 X	209 X	–
|assembly|	4,042,192	4,107,176	4,106,391
# contigs	275	2	2
% ANI	99.97815	99.94205	99.97385
**AMR (predicted from 82 *V. cholerae* genomes)**			
APH(3”)-Ib	74	74	74
APH(6)-Id	74	74	74
CRP	82	82	82
VC varG	79	79	79
almG	82	82	82
catB9	79	79	79
dfrA1	79	79	79
floR	74	74	74
rsmA	82	82	82
sul2	74	74	74
EC parE	82	35	82

*Displayed are average numbers over the 82 isolates. Details for each isolate can be viewed in Supplementary Table 1. Gene identifiers are the exact names used by CARD. # reads, average number of reads; |assembly|, average assembly length in nt; # contigs, median number of contigs; % ANI, average nucleotide identity; VC varG, Vibrio cholerae varG; EC parE, Escherichia coli parE conferring resistance to fluoroquinolones*.

The average accuracy of the assembled genomes was 99.942% for ONT MinION, 99.978% for Illumina, and 99.974% for the hybrid assembly. Therefore, when being interested in the absence or presence of genes, their genomic orientation, larger gene duplications and more importantly plasmids reconstructions, as desired for predicting AMR based on gene presence, we recommend using ONT MinION sequencing (Lemon et al., 2017; Golparian et al., 2018). Because plasmids are rare in pandemic *V. cholerae* strains (Jaskólska et al., 2022) we did not apply a particular library preparation method for recovery of plasmids in this study. However, when applying this method to other bacterial species, where small plasmids play a major role in conferring AMR, the ONT rapid library preparation method may be more suitable (Wick et al., 2021).

Contrary, when focusing on specific antibiotic agents, for which resistance phenotypes are associated with the presence of single nucleotide polymorphisms (SNPs), e.g., the fluoroquinolone Ciprofloxacin, we recommend Illumina sequencing (Dahl et al., 2021; Hodges et al., 2021). Currently, the ONT MinION sequencing produces too many errors for a reliable SNP analysis, however, with the upcoming R10 pore model of ONT MinION, we expect both sequencing methods to be equal in performance.

In summary, for the prediction of AMR, we conclude, that ONT MinION sequencing is similarly suitable for broad AMR studies as Illumina sequencing. However, ONT MinION sequencing brings the advantages of in-field studies, and in case a clinical sample is applied directly, can provide a faster result (refer below) and financial handling (refer below).

### 3.3. Antibiotic Resistance Genes Present in Illumina and Nanopore Sequencing

In total, Abricate and RGI detected ten different resistance-conferring genes against different antibiotics for the 82 *V. cholerae* isolates: *APH(3”)-Ib*, and *APH(6)-Id* confer resistance to aminoglycosides; *rsmA* against fluoroquinolones, diaminopyrimidines and phenicols; *CRP* against macrolides, fluoroquinolones, and penams; *dfrA1* against diaminopyrimidines; *floR* and *catB9* against phenicols; *Vibrio cholerae varG* against carbapenems; *almG* against polymyxins; and *sul2* against sulfonamides. There was no difference detected in Illumina and ONT MinION reads regarding the detection of AMR genes, refer to Table 1.

Several point mutations present in the gene *parE* in *V. cholerae* confer resistance against fluoroquinolones (Zhou et al., 2013) (EC *parE* in Table 1). Only RGI is able to detect this type of genetic variation. Accordingly, this resistance marker could be reliably detected in the accurate Illumina read based and in the hybrid assembly, whereas ONT reads this genetic variant was detected only in 35 of the 82 isolates analyzed.

### 3.4. Sequencing Can Replace AST for Five of Seven Antibiotics Tested

Independent of the chosen sequencing method, phenotypic AST can be replaced for most but not all antibiotical agents tested in this study. In the following, we present results from the hybrid assembly method for 80 isolates, for which both, sequencing as well as phenotypic AST data, were generated (refer to Section 2.2).

#### 3.4.1. Trimethoprim/Sulfamethoxazole (SxT)

In *V. cholerae*, the genes *dfrA1* and *sul2* are encoding trimethoprim and sulfamethoxazole resistance, respectively (Das et al., 2020). AST resulted in resistance to SxT for 77 isolates, and computationally, we were able to predict resistance correctly for 72 isolates, for which both genes were identified. In four of the five remaining phenotypically resistant isolates *dfrA1* was detected and *sul2* was missing, which should theoretically result in susceptibility of the bacterial isolates to SxT; and for one isolate, although resistant in the AST, none of the two genes was detected in the assembled genome. Three isolates were susceptible to SxT and CholerAegon correctly did not identify both genes, refer to Table 2 and for details Supplementary Table 4. Despite five discrepancies found we come to the conclusion that sequencing can replace AST testing for SxT, emphasizing that, in practice, the detection of either *dfrA1* or *sul2* could be considered sufficient to computationally classify a bacterial isolate as resistant. In these cases, in order to ensure treatment success, SxT would not be recommended for treatment.

**Table 2:** Numbers in boxes indicate the number of isolates, for which a gene (responsible for this phenotype) has been found.

	**SxT**	**NA**	**CIP**	**CN**	**TE**	**C**	**AMP**
**Genes**	**dfrA1 + sul2**	**EC parE**	**EC parE**	**var**	**var**	**catB9, floR**	**var**
S	0/3	17/17	18/18	0/80	0/80	77/80	0/20
SD	–	–	62/62	–	–	–	–
I	–	–	–	–	–	–	0/60
R	72/77	63/63	–	–	–	–	–
**Correct**	75	63	62	80	80	3	20
**False**	5	17	18	0	0	77	60
Seq?	YES	YES	YES	YES	YES	NO	NO

*Details can be found in the Supplementary Material. S, susceptible; SD, susceptible dose-dependent; R, resistant; I, intermediate; according to CLSI and EUCAST guidelines 2015 (see Section 2.2). Seq?, can sequencing replace AST testing?; AMP, ampicillin; C, chloramphenicol; CIP, ciprofloxacin; CN, Gentamicin A; TE, tetracycline; SxT, trimethoprimsulfamethoxazole; NA, nalidixic acid; EC parE, E. coli parE conferring resistance to fluoroquinolones; var, various genes*.

#### 3.4.2. Nalidixic Acid (NA), Ciprofloxacin (CIP)

In *V. cholerae* quinolone resistance is mediated by point mutations in the genes encoding Topoisomerase IV and DNA Gyrase (topoisomerase II) enzymes, *parCE* and *gyrAB*, respectively (De, 2021). An isolate exhibits resistance to NA or CIP when SNPs are present in these genes, protecting the bacterial enzyme from inhibition by the antibiotic agent (Aldred et al., 2014). For NA, AST resulted in 63 resistant and 17 susceptible isolates. With respect to CIP, the majority of isolates (62) appeared to be AB dose dependent susceptible, and 18 isolates were susceptible isolates. For all 80 isolates the D476N mutation in the EC *parE* gene has been found, providing evidence that the sole presence of this variant appears not sufficient to confer resistance to NA and CIP in approximately one quarter of our *V. cholerae* isolates. As a result, if AST was replaced by sequencing, for 63 and 62 of the 80 isolates a treatment with NA and CIP, respectively, would not be recommended, which is in accordance with the AST results. In the case of the susceptible 17 and 18 isolates, however, the NA/CIP treatment would not be recommended, although it would have been possible to manage the infection with these ABs.

#### 3.4.3. Gentamicin A (CN), Tetracycline (TE)

Differing resistance mechanisms to both aminoglycosides, CN and TE, have been described and the respective genes in *V. cholerae* are located on self-transmissable plasmids (Garneau-Tsodikova and Labby, 2016; Das et al., 2020). In our study, the phenotypic AST found in 80/80 and 80/80 isolates to be susceptible to CN and TE, respectively. In accordance with these *in vitro* findings, for both antibiotics no resistance-mediating gene was found when applying CholerAegon. We conclude that the prediction of the profile for these two ABs by sequencing is possible.

#### 3.4.4. Chloramphenicol (C)

Similar to the aminoglycoside antibiotics, a number of genes have been associated with resistance to C in *V. cholerae*, among them *catB9* and *florB*, encoding a C acetyl transferase (CAT) and a C efflux pump (Alcock et al., 2019; Das et al., 2020), respectively. AST has shown that all 80 isolates had a susceptible phenotype to C. Of these, CholerAegon identified three as susceptible, too. However, at least one of the two resistance genes *catB9* or *floR* was detected in 77 isolates, which—based on the genome analyses only—would have led to a resistant isolate. Although a medical treatment based on the sequencing results would not cause any harm to the patient (due to avoiding treatment with C), we would not recommend replacing AST with sequencing for C, because in almost all cases this treatment would have helped the patient.

#### 3.4.5. Ampicillin (AMP)

With regard to AMP, several genes are known to be responsible for AMR (Das et al., 2020). In the AST, 20 isolates have been found to be susceptible and 60 isolates have been found with an intermediate phenotype. Whereas for the 20 susceptible isolates the analysis of the genomes correctly did not reveal any resistance-associated gene, also no genetic resistance marker was found for the 60 intermediate phenotypes. In the case of AMP the AST intermediate phenotype could not be identified by the genome analysis. Possibly, the intermediate phenotypes maybe explained by resistance mechanisms that are independent of resistance genes, such as protein-regulatory mechanisms, which have been described in *V. cholerae* O1 strains resistant to ampicillin (Nguyen et al., 2009).

As described above, for NA, CIP, and C we observed discrepancies between the predicted *in silico* and *in vitro* AMR profiles. In these cases, *V. cholerae* isolates were found susceptible to AST, but resistance genes were found to be present. It is important to mention, that for phenotypic AST the classification into susceptible, resistant, dose-dependent and intermediate phenotype is solely defined by the breakpoints used. As a consequence, when applying different or updated breakpoints, as described in a recent study, correlation results may slightly differ (Opintan et al., 2021).

### 3.5. *Vibrio cholerae* Resistance Genes Visible After 40 min of Nanopore Sequencing

**Figure 2:**
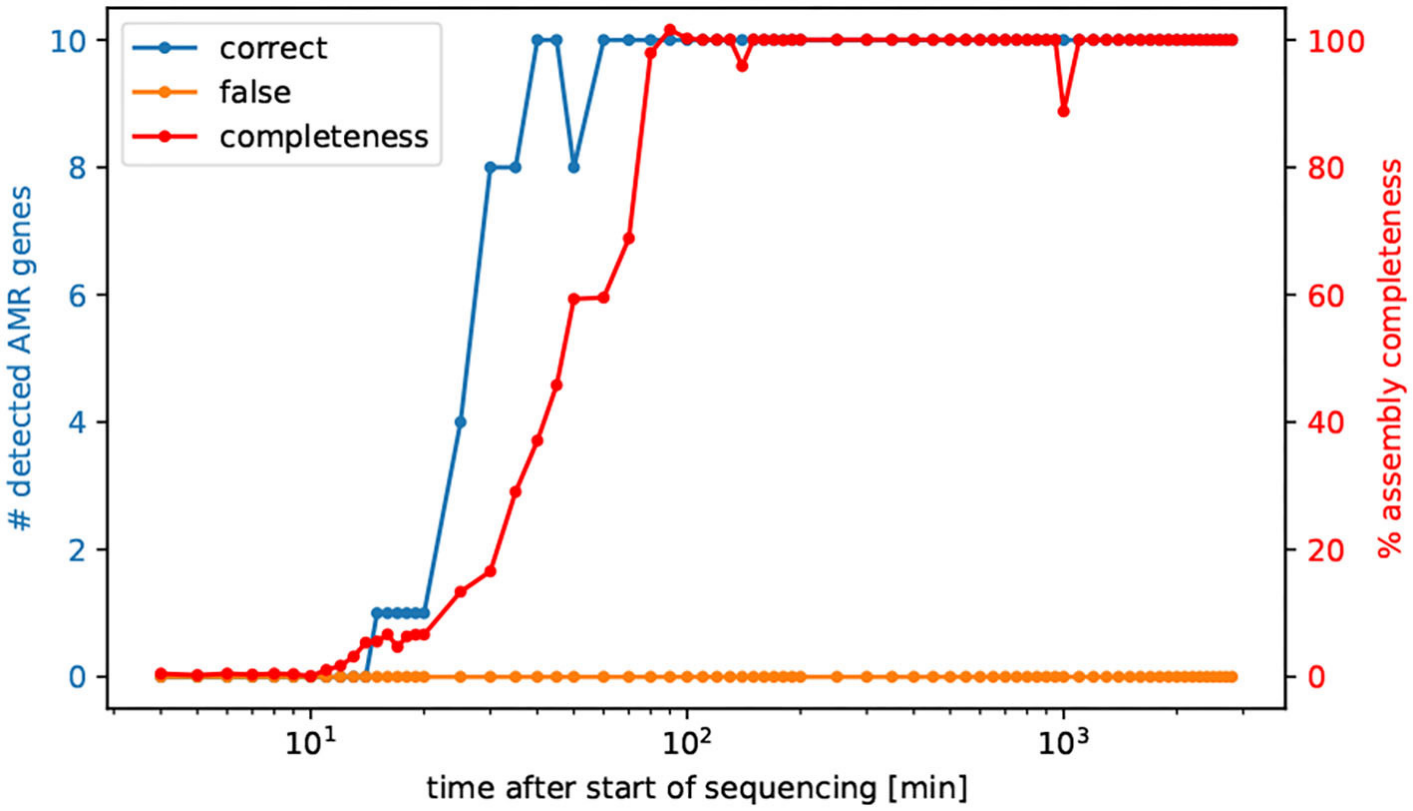
Number of detected AMR genes by the elapsed time of sequencing. We selected cumulative temporal subsets of the nanopore read data of isolate Iso02507. We used CholerAegon for assembly and subsequent AMR detection on these subsets. With 10 multiplexed isolates on this flowcell, all resistance genes from homolog models are detectable in the assembly after 60 min.

To successfully eliminate a pathogenic bacterial infection the time between diagnosis and initiation of treatment with an effective antibiotic drug is crucial. Hence, tracking the sequenced reads in minutes, we asked after how many minutes all AMR genes are detectable for the prediction of AMR, and after how much time of sequencing the entire genome is possible to assemble. Interestingly after already 60 min all AMR genes of *V. cholerae* are detectable in the same quality as the fully sequenced and assembled genomes, refer to Figure 2. More precisely, after already 40 min all AMR genes could be identified, however not in full length, refer to Supplementary Table 3.

For this study, we used bacterial isolates that had already undergone the selective culturing process and were used subsequently for DNA extraction and library preparation. In our case, the time needed for culturing was 1 day, and DNA extraction and library preparation took ~16 h. In order to effectively apply this method directly sequencing a clinical sample from a patient with acute watery diarrhea could speed up the process. But, because of the plethora of bacterial species present in a stool sample, this approach will likely require a higher sequencing depth.

### 3.6. Ninety-Six Bacteria of About 4 Mb Can Be Sequenced for About 30 € on ONT MinION

**Figure 3:**
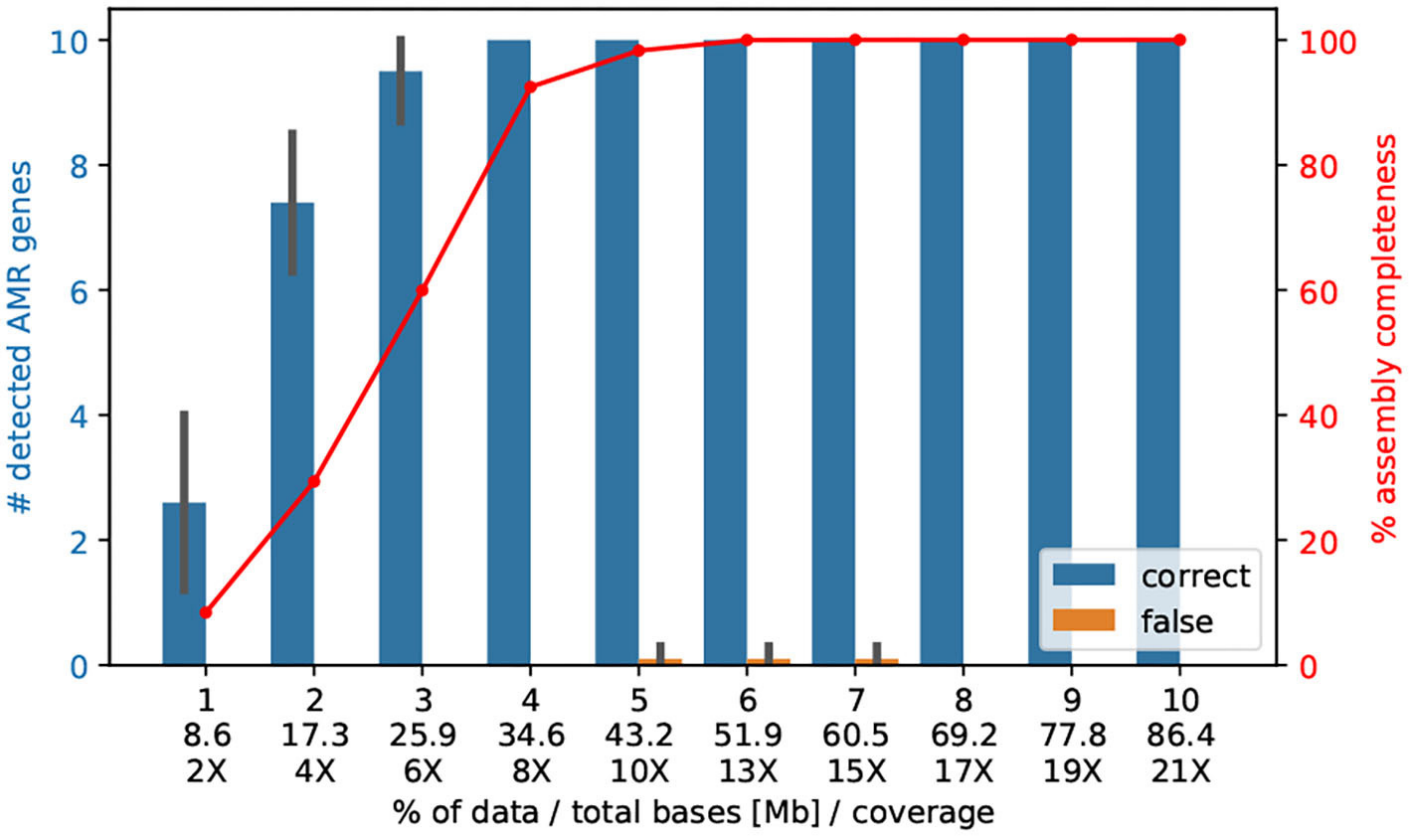
The number of AMR genes found with a randomly selected lower amount of Nanopore data. Here, we subsampled the Nanopore data (*n* = 10 replicates) of isolate Iso02507 at various percentages and performed assembly and AMR detection with CholerAegon for all subsets. About 4% (35 Mb) of the total Nanopore data (864 Mb) is enough to achieve the same detection result as using the full data for protein homolog models of AMR genes. This demonstrates that a throughput of ~35 Mb (8 X coverage of the 4.1 Mb genome) is sufficient for these models.

We sequenced 10–12 genomes of *V. cholerae* (~4.1 Mb) on one ONT MinION Flowcell and received an average coverage of 209 X, refer to Table 1. We raised the question if with a lower coverage we could also (i) assemble the entire genome correctly; and (ii) find the important AMR genes. In fact, already 4% of the reads (8.36 X) are sufficient enough to detect all 10 AMR genes^2^, and only 5% of the reads (43.2 Mb, 10X coverage) are necessary to assemble the entire genome correctly into the two chromosomes, refer to Figure 3.

By not aiming for the optimal cutoff of sequencing depth, but instead of allowing double coverage, we could bring 96 *V. cholerae* onto one ONT MinION Flowcell, resulting in costs of <40€ per sample with a comfortable coverage of 21 X to reconstruct the *V. cholera* genome, refer to Table 3. If we use primer-ligated barcoding, we reduce the costs of the barcoding procedure and can achieve a price of 31 per bacterial isolate with a genome size of 4.1 Mb.

**Table 3:** Sequencing costs in Euro (€) for Illumina and ONT per bacterial isolate (^*^) with an average genome length of 4.1 Mb for different sequencing strategies and resulting coverages.

	**Illumina**	**ONT**			
**Reagent**	**96 samples**	**1 sample**	**10 samples**	**96 samples**	**96 samples** ^(**)^
Ultrasound lysing	0	0	0	0	0
Phenol/ Chloroform	288	3	30	288	288
Qubit	96	1	10	96	96
Flowcell	1,102	545	545	545	545
Library	3,703	109	109	109	109
Barcoding	631	0	24	109	–
Enzyme	–	38	267	2,158	1,518
Qubit	–	3	34	288	100
Ampure	58	2	19	165	305
Total	5,878	701	1,058	3,758	2,961
Total/ Isolate^*^	61	701	105.8	39.15	30.84
Coverage/ Isolate^*^	49.54 X	2,090 X	209 X	21.77 X	21.77 X

(**) *Primer-ligated barcoding: 96 primers are ligated to 96 isolates and used as barcodes. Costs refer to current prices in Germany as of the time of publication*.

The cost calculation for Illumina sequencing (refer to Material and Methods section 2.4) was based on a maximum output of 19.5 Gb, whereas for ONT (refer to Material and Methods section 2.5) 8.6 Gb data has been generated. During the preparation of the manuscript, the new chemistry of the SQK-LSK110 ligation sequencing kit in conjugation with the R9.4.1 flowcell has improved the output capacity considerably (average output now: 18 Gb). Therefore, we can expect for 40€ even a coverage of 40 X, or lower the sequencing costs further per sample.

## 4. Conclusion

Here, we present CholerAegon, a bioinformatic pipeline for the prediction of bacterial AMR profiles from whole genome sequencing data. CholerAegon integrates long and short sequencing reads to produce genome assemblies and predicts an AMR profile based on a combined approach including the advantages of the two tools RGI and Abricate. By applying CholerAegon to *V. cholerae* sequences, we show that the prediction of antibiotic resistance profiles of *V. cholerae* works efficiently and with a comparable outcome for the Illumina and the ONT MinION sequencing platforms. When interested in specific SNPs as a resistance marker, we would recommend the less error-prone technology of Illumina. For the detection of larger genomic variations, which represent most resistance makers found in bacteria, the ONT MinION technology would preferably be used, because genome assemblies are significantly improved when using long reads.

Subsequently, the predicted AMR profiles based on sequencing data were correlated with phenotypic AST results for seven antibiotic agents for the same *V. cholerae* isolates. The correlation revealed, that in the case of *V. cholerae, in vitro* AST can be replaced by whole genome data analysis for the following antibiotics: Trimethoprim/Sulfamethoxazole, Nalidixic acid, Ciprofloxain, Chloramphenicol and Tetracycline but not for Chloramphenicol and Ampicillin.

With special emphasis on the applicability of this approach in areas where cholera outbreaks occur frequently, we analyzed time and costs of the sequencing for the portable ONT MinION platform. After 40 min of sequencing the coverage was sufficient in order to detect all resistance genes present in the *V. cholerae* genome. Additionally, by applying a custom-made barcoding strategy, we could reduce the sequencing costs to ~30€ to produce one 4.1 Mb-sized genome with sufficient coverage of 21X on the ONT MinION platform. In order to use the herein described approach as a standardized diagnostic tool in the future it will be necessary to establish specific criteria for ABs and organisms in question (e.g., minimum coverages) to ensure quality of sequencing data and reproducible results. Furthermore, the availability of curated AMR databases, in particular, species-centric databases with continuous integration of new resistance genes and mechanisms are key for this method.

The application will be particularly useful for strengthening AMR surveillance, at the individual, but also at the community level where it may be used to produce resistome profiles from metagenomes. Hence, genome sequencing, together with CholerAegon can be implemented widely, as part of a preparedness strategy to ensure that countries are ready to rapidly detect and respond to AMR in *V. cholerae* and other bacteria to reduce the spread of AMR.

## Data Availability Statement

Whole genome sequences have been deposited in the European Nucleotide Archive (ENA) at EMBL-EBI under accession number PRJEB51675 (https://www.ebi.ac.uk/ena/browser/view/PRJEB51675). CholerAegon is freely available at https://github.com/RaverJay/CholerAegon. Supplementary code used for writing this manuscript is available at https://github.com/RaverJay/CholerAegon/tree/main/paper.

## Author Contributions

VF, SK, SH-L, SW, DE, JM, MM, and KS conceived and designed the experiments. VF, SK, AS, DW, BL, and SH-L performed the experiments. SK and MM developed CholerAegon. SK, VF, TT, MM, and KS analyzed the data. VF, SK, MM, and KS wrote the manuscript. All authors contributed to the article and approved the submitted version.

## Conflict of Interest

The authors declare that the research was conducted in the absence of any commercial or financial relationships that could be construed as a potential conflict of interest.

## Publisher's Note

All claims expressed in this article are solely those of the authors and do not necessarily represent those of their affiliated organizations, or those of the publisher, the editors and the reviewers. Any product that may be evaluated in this article, or claim that may be made by its manufacturer, is not guaranteed or endorsed by the publisher.

## Footnotes

^1^The parameter –do_all_assemblies allows proceeding with all three assemblies.

^2^Ten AMR genes are defined as (1) APH(3”)-Ib; (2) APH(6)-Id; (3) CRP; (4) *V. cholerae* varG; (5) almG; (6) catB9; (7) dfrA1; (8) floR; (9) sul2; (10) rsmA.
