# Supplementary material for: Prediction of Antibiotic Susceptibility Profiles of Vibrio cholerae Isolates From Whole Genome Illumina and Nanopore Sequencing Data: CholerAegon
Source: Front Microbiol. 2022 Jun 22;13:909692. doi: 10.3389/fmicb.2022.909692 (PMC9257098; doi:10.3389/fmicb.2022.909692)
Supplement: Supplementary file 1 [file Table_1.pdf]

Table 1: General statistics of the isolate datasets and assemblies for long reads (LR), short reads (SR) and hybrid assembly (HY). #LR – number of long reads; MedLR – median length of long reads; #SR – number of short reads; LRN – number of contigs in long-reads-only assembly; LRL – length of long-reads-only assembly [nt]; HYN – number of contigs in hybrid assembly; HYL – length of hybrid assembly; SRN – number of contigs in short-reads-only assembly; SRL – length of short-reads-only assembly; LRcov – mean coverage of long reads against hybrid assembly; SRcov – mean coverage of short reads against hybrid assembly; LRANI – mean nucleotide identity of long-reads-only assembly; HYANI – mean nucleotide identity of hybrid assembly; SRANI – mean nucleotide identity of short-reads-only assembly; LRgenes – number of found AMR genes in long-reads-only assembly; HYgenes – number of found AMR genes in hybrid assembly; SRgenes – number of found AMR genes in short-reads-only assembly;

	Long reads			Short reads		Assembly statistics						Coverage		ANI			AMR genes		
Isolate	#LR	MedLR	Total bases LR	#SR	Total bases SR	LRN	LRL	HYN	HYL	SRN	SRL	LRcov	SRcov	LRANI	HYANI	SRANI	LRgenes	HYgenes	SRgenes
Iso02501	46640	6906	6.211781e+08	4539506	3.359234e+08	2	4152257	2	4153676	310	4064154	146.76	78.44	99.9352	99.9719	99.9801	11	11	11
Iso02502	292723	489	9.682356e+08	6220958	4.603509e+08	2	4156951	2	4157726	270	4082686	227.74	106.03	99.9379	99.9714	99.9821	11	11	11
Iso02503	83658	2078	6.788984e+08	6632348	4.907938e+08	2	4156280	2	4157109	292	4082841	160.25	113.07	99.9350	99.9651	99.9772	11	11	11
Iso02504	202399	665	1.051930e+09	8852870	6.551124e+08	2	4156971	2	4158025	257	4082430	247.74	147.21	99.9366	99.9720	99.9796	10	11	11
Iso02506	99643	1979	7.355847e+08	2286616	1.692096e+08	2	4139789	2	4141263	328	4073386	174.45	39.93	99.9389	99.9716	99.9777	6	7	7
Iso02507	80319	3335	8.643858e+08	12689044	9.389893e+08	2	4144502	2	4145239	238	4080641	204.10	203.02	99.9413	99.9713	99.9788	10	11	11
Iso02508	161724	6051	1.841609e+09	4833446	3.576750e+08	1	4148483	1	4150096	289	4080339	435.60	83.84	99.9487	99.9712	99.9780	11	11	11
Iso02509	87843	3106	9.349537e+08	3341418	2.472649e+08	2	4184926	2	4185953	315	4083618	218.74	57.71	99.9465	99.9756	99.9812	11	11	11
Iso02510	53733	2848	5.718420e+08	6853738	5.071766e+08	2	4171242	2	4172047	302	4083798	134.17	118.41	99.9349	99.9684	99.9815	11	11	11
Iso02511	87054	1744	6.072800e+08	7492692	5.544592e+08	2	4156988	2	4157777	283	4082632	142.65	128.81	99.9395	99.9660	99.9760	10	11	11
Iso02512	72274	3468	7.235894e+08	5008082	3.705981e+08	2	4138759	2	4139488	292	4082859	172.40	87.35	99.9401	99.9756	99.9788	10	11	11
Iso02513	135451	7448	1.974267e+09	6285480	4.651255e+08	2	4185029	2	4185917	265	4067818	465.35	108.34	99.9327	99.9684	99.9826	11	11	11
Iso02514	33611	13767	6.172206e+08	5844186	4.324698e+08	2	4138775	2	4140117	266	4081772	147.18	101.82	99.9387	99.9722	99.9777	10	11	11
Iso02515	63245	11722	1.105906e+09	6357126	4.704273e+08	2	4171231	2	4171927	280	4082904	261.62	108.47	99.9377	99.9703	99.9791	11	11	11
Iso02517	94491	5997	1.292397e+09	5681770	4.204510e+08	2	4171166	2	4171968	274	4082949	305.59	98.14	99.9424	99.9738	99.9758	10	11	11
Iso02518	51468	2842	4.937027e+08	3440104	2.545677e+08	2	4119362	2	4120156	235	4059088	118.21	59.28	99.9288	99.9709	99.9781	11	11	11
Iso02519	77765	2631	6.104780e+08	5739368	4.247132e+08	2	4082145	2	4082879	273	4025879	147.39	100.89	99.9410	99.9717	99.9809	7	7	7
Iso02520	130666	1337	1.030614e+09	6685926	4.947585e+08	1	4156698	1	4157414	261	4081419	244.79	115.90	99.9379	99.9737	99.9756	11	11	11
Iso02522	97019	3469	9.935001e+08	6194856	4.584193e+08	2	4107587	2	4108283	279	4035295	238.91	108.55	99.9417	99.9715	99.9821	10	11	11
Iso02523	25483	17681	5.341279e+08	7668808	5.674918e+08	2	4093430	2	4094252	279	4246238	128.86	131.45	99.9387	99.9703	99.9842	10	11	11
Iso02524	42443	11416	6.965869e+08	6623884	4.901674e+08	2	4093420	2	4094158	271	4034365	167.92	115.06	99.9438	99.9748	99.9832	11	11	11
Iso02525	80222	6477	1.010185e+09	8368452	6.192654e+08	2	4102147	2	4102833	289	4036140	242.94	146.05	99.9463	99.9757	99.9817	10	11	11
Iso02527	50034	8599	6.723040e+08	8341548	6.172746e+08	2	4093437	2	4094150	306	4036660	162.03	146.89	99.9424	99.9765	99.9821	10	11	11
Iso02529	49364	11216	7.920022e+08	5286258	3.911831e+08	2	4102026	2	4102875	287	4035247	190.48	93.29	99.9426	99.9742	99.9823	10	11	11
Iso02532	137875	3440	1.163668e+09	3920884	2.901454e+08	2	4102946	2	4103611	345	4038605	279.69	68.92	99.9439	99.9766	99.9767	10	11	11
Iso02533	102735	3693	8.865780e+08	7412268	5.485078e+08	2	4098101	2	4098831	272	4035286	213.29	118.31	99.9439	99.9772	99.9773	10	11	11
Iso02534	113909	2330	9.399045e+08	6683336	4.945669e+08	2	4098043	2	4099091	299	4036527	226.29	117.39	99.9427	99.9722	99.9780	11	11	11
Iso02535	104816	1501	7.637277e+08	4819916	3.566738e+08	2	4098110	2	4098833	286	4036027	183.56	82.46	99.9433	99.9779	99.9800	10	11	11
Iso02536	109444	1517	7.493810e+08	5986052	4.429678e+08	2	4092536	2	4093252	286	4036213	180.50	100.38	99.9410	99.9752	99.9776	10	11	11
Iso02537	87178	4125	9.234052e+08	4493460	3.325160e+08	2	4079110	2	4079774	336	4031298	222.51	79.61	99.9453	99.9757	99.9789	11	11	11
Iso02538	150937	5950	1.691496e+09	3265476	2.416452e+08	2	3891205	2	3891872	433	3821731	427.64	60.77	97.9727	97.9891	98.0883	4	4	4
Iso02539	179287	1853	1.460921e+09	6041386	4.470626e+08	3	4087067	3	4086829	526	4026832	351.95	96.89	98.0841	98.1003	98.1544	4	4	4
Iso02541	67996	8368	9.311779e+08	6514738	4.820906e+08	2	4108386	2	4109318	303	4032819	222.83	106.41	99.9465	99.9815	99.9777	11	11	11
Iso02542	62043	6487	8.015435e+08	4749958	3.514969e+08	2	4098008	2	4099105	292	4036473	192.11	80.52	99.9419	99.9727	99.9778	10	11	11
Iso02544	111848	3571	1.003935e+09	6883562	5.093836e+08	2	4098091	2	4098726	269	4036232	240.96	109.36	99.9399	99.9722	99.9794	11	11	11
Iso02545	67715	10546	1.064973e+09	5428360	4.016986e+08	2	4098109	2	4098814	262	4035277	255.97	93.47	99.9454	99.9775	99.9796	11	11	11
Iso02546	67927	5939	7.623128e+08	6179906	4.573130e+08	2	4112358	2	4113063	276	4036254	182.17	98.04	99.9426	99.9780	99.9776	11	11	11
Iso02547	38284	10163	5.833681e+08	5765828	4.266713e+08	2	4098078	2	4098864	268	4036890	139.95	92.62	99.9429	99.9798	99.9777	10	11	11
Iso02549	80199	6552	1.006297e+09	5197720	3.846313e+08	2	4098140	2	4099180	294	4036508	241.92	89.13	99.9381	99.9749	99.9830	10	11	11
Iso02550	165740	3754	1.271312e+09	6313000	4.671620e+08	2	4098090	2	4098767	269	4034455	305.32	101.77	99.9422	99.9747	99.9781	11	11	11
Iso02551	64018	6102	7.639976e+08	6972138	5.130019e+08	2	4086912	2	4087743	264	4025182	184.18	111.36	99.9410	99.9733	99.9775	6	7	7
Iso02552	100663	5315	1.081667e+09	7259922	5.341712e+08	2	4099335	2	4100096	275	4035794	259.81	119.62	99.9340	99.9702	99.9792	11	11	11
Iso02553	122408	4831	1.280058e+09	5991662	4.408301e+08	2	4098091	2	4098838	307	4037466	307.87	104.57	99.9410	99.9731	99.9797	11	11	11
Iso02554	111962	6511	1.389001e+09	7080372	5.209121e+08	2	4099394	2	4100142	280	4036144	333.83	111.89	99.9408	99.9739	99.9787	11	11	11
Iso02555	169095	3350	1.238627e+09	6261926	4.607248e+08	2	4098088	2	4098836	275	4034471	297.30	106.08	99.9405	99.9753	99.9765	10	11	11
Iso02556	65092	9115	9.114845e+08	13590986	9.999437e+08	2	4092573	2	4093466	298	4036611	219.28	233.25	99.9401	99.9735	99.9781	11	11	11
Iso02557	98954	4818	1.027227e+09	8480046	6.275234e+08	2	4072752	2	4073467	266	4020914	248.17	145.78	99.9430	99.9729	99.9804	10	11	11
Iso02558	96726	6365	1.035210e+09	16150834	1.195162e+09	2	4093591	2	4094469	274	4035258	247.88	254.16	99.9435	99.9724	99.9773	10	11	11
Iso02559	71855	7236	9.486003e+08	6876062	5.088286e+08	2	4075668	2	4076344	253	4021263	230.05	110.96	99.9468	99.9790	99.9833	10	11	11
Iso02560	61854	4355	5.188199e+08	8195136	6.064401e+08	2	4092371	2	4093434	279	4035665	125.15	132.76	99.9409	99.9743	99.9746	10	11	11
Iso02561	37968	4856	3.839189e+08	7307386	5.407466e+08	2	4098088	2	4098992	281	4035874	92.53	118.63	99.9394	99.9820	99.9775	10	11	11
Iso02563	133570	8869	1.444448e+09	8937582	6.613811e+08	2	4092474	2	4093063	426	4043752	347.81	139.05	99.9461	99.9768	99.9793	10	11	11
Iso02564	24950	9130	3.617118e+08	5788050	4.283157e+08	2	4094498	2	4095446	265	4033706	87.27	90.88	99.9360	99.9714	99.9774	10	11	11
Iso02565	18980	12890	3.298826e+08	9612760	7.113442e+08	2	4096286	2	4097279	267	4031536	79.64	157.46	99.9336	99.9736	99.9782	10	11	11
Iso02566	47448	13045	8.468415e+08	6930672	5.128697e+08	2	4095018	2	4095709	274	4032966	204.40	108.12	99.9401	99.9734	99.9775	10	11	11
Iso02567	33464	8945	4.703223e+08	6091410	4.507643e+08	2	4098069	2	4098934	294	4036318	113.44	96.88	99.9457	99.9758	99.9784	11	11	11

Iso02568	46848	7276	5.088007e+08	7633332	5.648666e+08	2	4075660	2	4076284	289	4026135	122.81	127.40	99.9490	99.9769	99.9743	6	7	7
Iso02569	47794	8612	6.850451e+08	6216560	4.600254e+08	2	4098040	2	4098783	268	4035180	165.18	104.68	99.9409	99.9756	99.9774	10	11	11
Iso02570	27679	7082	3.736808e+08	8089588	5.986295e+08	2	4098081	2	4098946	275	4035554	90.05	135.65	99.9367	99.9734	99.9791	11	11	11
Iso02571	50609	3364	3.626072e+08	6569494	4.861426e+08	2	4112357	2	4113282	268	4034865	86.98	111.26	99.9364	99.9747	99.9792	10	11	11
Iso02572	192695	2538	9.550747e+08	4949896	3.662923e+08	3	4112121	3	4112671	272	4035212	228.30	83.70	99.9486	99.9756	99.9738	10	11	11
Iso02573	73716	3592	5.856306e+08	5812592	4.301318e+08	2	4099707	2	4100314	265	4034690	140.36	98.67	99.9385	99.9677	99.9743	11	11	11
Iso02575	67613	2862	4.891254e+08	9157646	6.776658e+08	5	4087902	5	4088837	262	4025368	117.68	145.62	99.9483	99.9771	99.9774	7	7	7
Iso02577	51976	8422	7.457054e+08	8292586	6.136514e+08	2	4112361	2	4113036	272	4035793	178.87	129.72	99.9478	99.9806	99.9762	10	11	11
Iso02579	71904	2280	5.090767e+08	8747308	6.473008e+08	2	4099886	2	4100825	282	4035920	122.21	138.38	99.9445	99.9747	99.9760	11	11	11
Iso02580	52840	5042	5.299890e+08	7106932	5.259130e+08	5	4099980	5	4100757	287	4036185	127.18	115.61	99.9411	99.9709	99.9752	10	11	11
Iso02581	90012	3183	6.752872e+08	6860678	5.076902e+08	2	4091862	2	4092845	290	4035849	162.53	109.12	99.9485	99.9792	99.9754	10	11	11
Iso02582	51791	7174	6.283331e+08	8490498	6.282969e+08	2	4098097	2	4098743	267	4034755	151.11	131.50	99.9417	99.9744	99.9834	10	11	11
Iso02583	200877	2544	1.446992e+09	8691446	6.431670e+08	2	4020144	2	4020216	406	3931627	354.73	145.65	97.9168	97.9259	98.0757	4	4	4
Iso02584	96786	3178	7.462824e+08	6638166	4.912243e+08	2	4098087	2	4098738	269	4035780	178.95	109.28	99.9458	99.9771	99.9778	10	11	11
Iso02585	37870	7471	4.802244e+08	9158080	6.776979e+08	2	4086812	2	4087480	257	4034437	115.44	149.36	99.9467	99.9731	99.9791	11	11	11
Iso02586	93789	2599	6.235416e+08	8667276	6.413784e+08	2	4098080	2	4098743	255	4035135	149.63	129.02	99.9437	99.9733	99.9829	10	11	11
Iso02587	141114	2436	1.234193e+09	7598210	5.622675e+08	2	4095379	2	4095978	259	4034704	298.02	121.70	99.9438	99.9723	99.9829	11	11	11
Iso02588	105526	4583	1.173648e+09	8135922	6.020582e+08	2	4098085	2	4098725	288	4036022	283.23	117.95	99.9528	99.9749	99.9789	10	11	11
Iso02589	188037	1121	1.193843e+09	7252936	5.367173e+08	2	4150021	2	4150623	251	4035052	284.32	112.54	99.9443	99.9738	99.9770	10	11	11
Iso02590	198456	3685	1.753775e+09	8056490	5.961803e+08	2	4098113	2	4098731	281	4035714	423.28	118.88	99.9486	99.9750	99.9810	10	11	11
Iso02591	102194	10725	1.642842e+09	6408482	4.742277e+08	2	4092384	2	4093385	266	4035874	397.17	109.23	99.9456	99.9775	99.9805	11	11	11
Iso02592	276625	943	1.860757e+09	16173092	1.196809e+09	2	4099414	2	4100025	254	4034644	448.88	255.57	99.9428	99.9678	99.9776	10	11	11
Iso02593	79713	13590	1.444008e+09	5226350	3.867499e+08	2	4098088	2	4098761	293	4035393	348.74	82.16	99.9447	99.9774	99.9818	10	11	11
Iso02594	56544	10753	9.164662e+08	8986550	6.650047e+08	2	4098150	2	4098842	272	4035125	221.22	133.59	99.9458	99.9766	99.9796	10	11	11
Iso02595	126549	7716	1.674081e+09	8042816	5.951684e+08	2	4112138	2	4112770	269	4035171	402.56	129.03	99.9519	99.9748	99.9775	10	11	11
Iso02597	53991	7451	7.398588e+08	9100442	6.734327e+08	3	4129969	3	4130637	249	4034126	177.11	138.59	99.9476	99.9790	99.9810	11	11	11
