# Supplementary material for: Prediction of Antibiotic Susceptibility Profiles of Vibrio cholerae Isolates From Whole Genome Illumina and Nanopore Sequencing Data: CholerAegon
Source: Front Microbiol. 2022 Jun 22;13:909692. doi: 10.3389/fmicb.2022.909692 (PMC9257098; doi:10.3389/fmicb.2022.909692)
Supplement: Supplementary file 2 [file Table_2.pdf]

Table 2: Antimicrobial resistance genes found in isolates of *V. cholerae*. We used CholerAegon to assemble 82 isolates and subsequently detect resistance genes. Numbers in cells belonging to AMR gene columns represent the % coverage of the reference gene sequence from the database (multiple numbers represent gene copies). ASM – assembly method; ANI – average nucleotide identity; NG – number of genes found; EC parE – Escherichia coli parE conferring resistance to fluoroquinolones; VC varG – Vibrio cholerae varG;

Isolate	ASM	ANI %	NG	APH(3")-Ib	APH(6)-Id	CRP	EC parE	VC varG	almG	catB9	dfrA1	floR	rsmA	sul2
Iso02501	hybrid	99.9719	11	100.0	100.0	100.0	99.37	100.0	100.0	100.0	100.0	100.0	106.56	100.0
Iso02501	longreads	99.9352	11	100.0	100.0	100.0	99.37	100.0	100.0	100.0	100.0	100.0	106.56	100.0
Iso02501	shortreads	99.9801	11	100.0	100.0	100.0	99.37	100.0	100.0	100.0	100.0	100.0	106.56	100.0
Iso02502	hybrid	99.9714	11	100.0	100.0	100.0	99.37	100.0	100.0	100.0	100.0	100.0	106.56	100.0
Iso02502	longreads	99.9379	11	100.0	100.0	100.0	99.37	100.0	100.0	100.0	100.0	100.0	106.56	100.0
Iso02502	shortreads	99.9821	11	100.0	100.0	100.0	99.37	100.0	100.0	100.0	100.0	100.0	106.56	100.0
Iso02503	hybrid	99.9651	11	100.0	100.0	100.0	99.37	100.0	100.0	100.0	100.0	100.0	106.56	100.0
Iso02503	longreads	99.9350	11	100.0	100.0	100.0	99.37	100.0	100.0	100.0	100.0	100.0	106.56	100.0
Iso02503	shortreads	99.9772	11	100.0	100.0	100.0	99.37	100.0	100.0	100.0	100.0	100.0	106.56	100.0
Iso02504	hybrid	99.9720	11	100.0	100.0	100.0	99.37	100.0	100.0	100.0	100.0	100.0	106.56	100.0
Iso02504	longreads	99.9366	10	100.0	100.0	100.0	.	100.0	100.0	100.0	100.0	100.0	106.56	100.0
Iso02504	shortreads	99.9796	11	100.0	100.0	100.0	99.37	100.0	100.0	100.0	100.0	100.0	106.56	100.0
Iso02506	hybrid	99.9716	7	.	.	100.0	99.37	100.0	100.0	100.0	100.0	.	106.56	.
Iso02506	longreads	99.9389	6	.	.	100.0	.	100.0	100.0	100.0	100.0	.	106.56	.
Iso02506	shortreads	99.9777	7	.	.	100.0	99.37	100.0	100.0	100.0	100.0	.	106.56	.
Iso02507	hybrid	99.9713	11	100.0	100.0	100.0	99.37	100.0	100.0	100.0	100.0	100.0	106.56	100.0
Iso02507	longreads	99.9413	10	100.0	100.0	100.0	.	100.0	100.0	100.0	100.0	100.0	106.56	100.0
Iso02507	shortreads	99.9788	11	100.0	100.0	100.0	99.37	100.0	100.0	100.0	100.0	100.0	106.56	100.0
Iso02508	hybrid	99.9712	11	100.0	100.0	100.0	99.37	100.0	100.0	100.0	100.0	100.0	106.56	100.0
Iso02508	longreads	99.9487	11	100.0	100.0	100.0	99.37	100.0	100.0	100.0	100.0	100.0	106.56	100.0
Iso02508	shortreads	99.9780	11	100.0	100.0	100.0	99.37	100.0	100.0	100.0	100.0	100.0	106.56	100.0
Iso02509	hybrid	99.9756	11	100.0	100.0	100.0	99.37	100.0	100.0	100.0	100.0	100.0	106.56	100.0
Iso02509	longreads	99.9465	11	100.0	100.0	100.0	99.37	100.0	100.0	100.0	100.0	100.0	106.56	100.0
Iso02509	shortreads	99.9812	11	100.0	100.0	100.0	99.37	100.0	100.0	100.0	100.0	100.0	106.56	100.0
Iso02510	hybrid	99.9684	11	100.0	100.0	100.0	99.37	100.0	100.0	100.0	100.0	100.0	106.56	100.0
Iso02510	longreads	99.9349	11	100.0	100.0	100.0	99.37	100.0	100.0	100.0	100.0	100.0	106.56	100.0
Iso02510	shortreads	99.9815	11	100.0	100.0	100.0	99.37	100.0	100.0	100.0	100.0	100.0	106.56	100.0
Iso02511	hybrid	99.9660	11	100.0	100.0	100.0	99.37	100.0	100.0	100.0	100.0	100.0	106.56	100.0
Iso02511	longreads	99.9395	10	100.0	100.0	100.0	.	100.0	100.0	100.0	100.0	100.0	106.56	100.0
Iso02511	shortreads	99.9760	11	100.0	100.0	100.0	99.37	100.0	100.0	100.0	100.0	100.0	106.56	100.0
Iso02512	hybrid	99.9756	11	100.0	100.0	100.0	99.37	100.0	100.0	100.0	100.0	100.0	106.56	100.0
Iso02512	longreads	99.9401	10	100.0	100.0	100.0	.	100.0	100.0	100.0	100.0	100.0	106.56	100.0
Iso02512	shortreads	99.9788	11	100.0	100.0	100.0	99.37	100.0	100.0	100.0	100.0	100.0	106.56	100.0
Iso02513	hybrid	99.9684	11	100.0	100.0	100.0	99.37	100.0	100.0	100.0	100.0	100.0	106.56	100.0
Iso02513	longreads	99.9327	11	100.0	100.0	100.0	99.37	100.0	100.0	100.0	100.0	100.0	106.56	100.0
Iso02513	shortreads	99.9826	11	100.0	100.0	100.0	99.37	100.0	100.0	100.0	100.0	100.0	106.56	100.0
Iso02514	hybrid	99.9722	11	100.0	100.0	100.0	99.37	100.0	100.0	100.0	100.0	100.0	106.56	100.0
Iso02514	longreads	99.9387	10	100.0	100.0	100.0	.	100.0	100.0	100.0	100.0	100.0	106.56	100.0
Iso02514	shortreads	99.9777	11	100.0	100.0	100.0	99.37	100.0	100.0	100.0	100.0	100.0	106.56	100.0
Iso02515	hybrid	99.9703	11	100.0	100.0	100.0	99.37	100.0	100.0	100.0	100.0	100.0	106.56	100.0
Iso02515	longreads	99.9377	11	100.0	100.0	100.0	99.37	100.0	100.0	100.0	100.0	100.0	106.56	100.0
Iso02515	shortreads	99.9791	11	100.0	100.0	100.0	99.37	100.0	100.0	100.0	100.0	100.0	106.56	100.0
Iso02517	hybrid	99.9738	11	100.0	100.0	100.0	99.37	100.0	100.0	100.0	100.0	100.0	106.56	100.0
Iso02517	longreads	99.9424	10	100.0	100.0	100.0	.	100.0	100.0	100.0	100.0	100.0	106.56	100.0
Iso02517	shortreads	99.9758	11	100.0	100.0	100.0	99.37	100.0	100.0	100.0	100.0	100.0	106.56	100.0
Iso02518	hybrid	99.9709	11	100.0	100.0	100.0	99.37	100.0	100.0	100.0	100.0	100.0	106.56	100.0
Iso02518	longreads	99.9288	11	100.0	100.0	100.0	99.37	100.0	100.0	100.0	100.0	100.0	106.56	100.0
Iso02518	shortreads	99.9781	11	100.0	100.0	100.0	99.37	100.0	100.0	100.0	100.0	100.0	106.56	100.0
Iso02519	hybrid	99.9717	7	.	.	100.0	99.37	100.0	100.0	100.0	100.0	.	106.56	.
Iso02519	longreads	99.9410	7	.	.	100.0	99.37	100.0	100.0	100.0	100.0	.	106.56	.
Iso02519	shortreads	99.9809	7	.	.	100.0	99.37	100.0	100.0	100.0	100.0	.	106.56	.
Iso02520	hybrid	99.9737	11	100.0	100.0	100.0	99.37	100.0	100.0	100.0	100.0	100.0	106.56	100.0
Iso02520	longreads	99.9379	11	100.0	100.0	100.0	99.37	100.0	100.0	100.0	100.0	100.0	106.56	100.0
Iso02520	shortreads	99.9756	11	100.0	100.0	100.0	99.37	100.0	100.0	100.0	100.0	100.0	106.56	100.0
Iso02522	hybrid	99.9715	11	100.0	100.0	100.0	99.37	100.0	100.0	100.0	100.0	100.0	106.56	100.0
Iso02522	longreads	99.9417	10	100.0	100.0	100.0	.	100.0	100.0	100.0	100.0	100.0	106.56	100.0
Iso02522	shortreads	99.9821	11	100.0	100.0	100.0	99.37	100.0	100.0	100.0	100.0	100.0	106.56	100.0
Iso02523	hybrid	99.9703	11	100.0	100.0	100.0	99.37	100.0	100.0	100.0	100.0	100.0	106.56	100.0
Iso02523	longreads	99.9387	10	100.0	100.0	100.0	.	100.0	100.0	100.0	100.0	100.0	106.56	100.0
Iso02523	shortreads	99.9842	11	100.0	100.0	100.0; 100.0	99.37	100.0	100.0	100.0	100.0	100.0	106.56	100.0
Iso02524	hybrid	99.9748	11	100.0	100.0	100.0	99.37	100.0	100.0	100.0	100.0	100.0	106.56	100.0
Iso02524	longreads	99.9438	11	100.0	100.0	100.0	99.37	100.0	100.0	100.0	100.0	100.0	106.56	100.0
Iso02524	shortreads	99.9832	11	100.0	100.0	100.0	99.37	100.0	100.0	100.0	100.0	100.0	106.56	100.0
Iso02525	hybrid	99.9757	11	100.0	100.0	100.0	99.37	100.0	100.0	100.0	100.0	100.0	106.56	100.0
Iso02525	longreads	99.9463	10	100.0	100.0	100.0	.	100.0	100.0	100.0	100.0	100.0	106.56	100.0
Iso02525	shortreads	99.9817	11	100.0	100.0	100.0	99.37	100.0	100.0	100.0	100.0	100.0	106.56	100.0
Iso02527	hybrid	99.9765	11	100.0	100.0	100.0	99.37	100.0	100.0	100.0	100.0	100.0	106.56	100.0
Iso02527	longreads	99.9424	10	100.0	100.0	100.0	.	100.0	100.0	100.0	100.0	100.0	106.56	100.0
Iso02527	shortreads	99.9821	11	100.0	100.0	100.0	99.37	100.0	100.0	100.0	100.0	100.0	106.56	100.0
Iso02529	hybrid	99.9742	11	100.0	100.0	100.0	99.37	100.0	100.0	100.0	100.0	100.0	106.56	100.0
Iso02529	longreads	99.9426	10	100.0	100.0	100.0	.	100.0	100.0	100.0	100.0	100.0	106.56	100.0
Iso02529	shortreads	99.9823	11	100.0	100.0	100.0	99.37	100.0	100.0	100.0	100.0	100.0	106.56	100.0
Iso02532	hybrid	99.9766	11	100.0	100.0	100.0	99.37	100.0	100.0	100.0	100.0	100.0	106.56	100.0
Iso02532	longreads	99.9439	10	100.0	100.0	100.0	.	100.0	100.0	100.0	100.0	100.0	106.56	100.0
Iso02532	shortreads	99.9767	11	100.0	100.0	100.0	99.37	100.0	100.0	100.0	100.0	100.0	106.56	100.0
Iso02533	hybrid	99.9772	11	100.0	100.0	100.0	99.37	100.0	100.0	100.0	100.0	100.0	106.56	100.0
Iso02533	longreads	99.9439	10	100.0	100.0	100.0	.	100.0	100.0	100.0	100.0	100.0	106.56	100.0
Iso02533	shortreads	99.9773	11	100.0	100.0	100.0	99.37	100.0	100.0	100.0	100.0	100.0	106.56	100.0
Iso02534	hybrid	99.9722	11	100.0	100.0	100.0	99.37	100.0	100.0	100.0	100.0	100.0	106.56	100.0
Iso02534	longreads	99.9427	11	100.0	100.0	100.0	99.37	100.0	100.0	100.0	100.0	100.0	106.56	100.0
Iso02534	shortreads	99.9780	11	100.0	100.0	100.0	99.37	100.0	100.0	100.0	100.0	100.0	106.56	100.0
Iso02535	hybrid	99.9779	11	100.0	100.0	100.0	99.37	100.0	100.0	100.0	100.0	100.0	106.56	100.0
Iso02535	longreads	99.9433	10	100.0	100.0	100.0	.	100.0	100.0	100.0	100.0	100.0	106.56	100.0
Iso02535	shortreads	99.9800	11	100.0	100.0	100.0	99.37	100.0	100.0	100.0	100.0	100.0	106.56	100.0
Iso02536	hybrid	99.9752	11	100.0	100.0	100.0	99.37	100.0	100.0	100.0	100.0	100.0	106.56	100.0
Iso02536	longreads	99.9410	10	100.0	100.0	100.0	.	100.0	100.0	100.0	100.0	100.0	106.56	100.0
Iso02536	shortreads	99.9776	11	100.0	100.0	100.0	99.37	100.0	100.0	100.0	100.0	100.0	106.56	100.0
Iso02537	hybrid	99.9757	11	100.0	100.0	100.0	99.37	100.0	100.0	100.0	100.0	100.0	106.56	100.0
Iso02537	longreads	99.9453	11	100.0	100.0	100.0	99.37	100.0	100.0	100.0	100.0	100.0	106.56	100.0
Iso02537	shortreads	99.9789	11	100.0	100.0	100.0	99.37	100.0	100.0	100.0	100.0	100.0	106.56	100.0
Iso02538	hybrid	97.9891	4	.	.	100.0	99.37	.	100.0	.	.	.	106.56	.
Iso02538	longreads	97.9727	4	.	.	100.0	99.37	.	100.0	.	.	.	106.56	.
Iso02538	shortreads	98.0883	4	.	.	100.0	99.37	.	100.0	.	.	.	106.56	.
Iso02539	hybrid	98.1003	4	.	.	100.0	99.37	.	100.0	.	.	.	106.56	.

Iso02539	longreads	98.0841	4	.	.	100.0	99.37	.	100.0	.	.	.	106.56	.
Iso02539	shortreads	98.1544	4	.	.	100.0	99.37	.	100.0	.	.	.	106.56	.
Iso02541	hybrid	99.9815	11	100.0	100.0	100.0	99.37	100.0	100.0	100.0	100.0	100.0	106.56	100.0
Iso02541	longreads	99.9465	11	100.0	100.0	100.0	99.37	100.0	100.0	100.0	100.0	100.0	106.56	100.0
Iso02541	shortreads	99.9777	11	100.0	100.0	100.0	99.37	100.0	100.0	100.0	100.0	100.0	106.56	100.0
Iso02542	hybrid	99.9727	11	100.0	100.0	100.0	99.37	100.0	100.0	100.0	100.0	100.0	106.56	100.0
Iso02542	longreads	99.9419	10	100.0	100.0	100.0	.	100.0	100.0	100.0	100.0	100.0	106.56	100.0
Iso02542	shortreads	99.9778	11	100.0	100.0	100.0	99.37	100.0	100.0	100.0	100.0	100.0	106.56	100.0
Iso02544	hybrid	99.9722	11	100.0	100.0	100.0	99.37	100.0	100.0	100.0	100.0	100.0	106.56	100.0
Iso02544	longreads	99.9399	11	100.0	100.0	100.0	99.37	100.0	100.0	100.0	100.0	100.0	106.56	100.0
Iso02544	shortreads	99.9794	11	100.0	100.0	100.0	99.37	100.0	100.0	100.0	100.0	100.0	106.56	100.0
Iso02545	hybrid	99.9775	11	100.0	100.0	100.0	99.37	100.0	100.0	100.0	100.0	100.0	106.56	100.0
Iso02545	longreads	99.9454	11	100.0	100.0	100.0	99.37	100.0	100.0	100.0	100.0	100.0	106.56	100.0
Iso02545	shortreads	99.9796	11	100.0	100.0	100.0	99.37	100.0	100.0	100.0	100.0	100.0	106.56	100.0
Iso02546	hybrid	99.9780	11	100.0	100.0	100.0	99.37	100.0	100.0	100.0	100.0	100.0	106.56	100.0
Iso02546	longreads	99.9426	11	100.0	100.0	100.0	99.37	100.0	100.0	100.0	100.0	100.0	106.56	100.0
Iso02546	shortreads	99.9776	11	100.0	100.0	100.0	99.37	100.0	100.0	100.0	100.0	100.0	106.56	100.0
Iso02547	hybrid	99.9798	11	100.0	100.0	100.0	99.37	100.0	100.0	100.0	100.0	100.0	106.56	100.0
Iso02547	longreads	99.9429	10	100.0	100.0	100.0	.	100.0	100.0	100.0	100.0	100.0	106.56	100.0
Iso02547	shortreads	99.9777	11	100.0	100.0	100.0	99.37	100.0	100.0	100.0	100.0	100.0	106.56	100.0
Iso02549	hybrid	99.9749	11	100.0	100.0	100.0	99.37	100.0	100.0	100.0	100.0	100.0	106.56	100.0
Iso02549	longreads	99.9381	10	100.0	100.0	100.0	.	100.0	100.0	100.0	100.0	100.0	106.56	100.0
Iso02549	shortreads	99.9830	11	100.0	100.0	100.0	99.37	100.0	100.0	100.0	100.0	100.0	106.56	100.0
Iso02550	hybrid	99.9747	11	100.0	100.0	100.0	99.37	100.0	100.0	100.0	100.0	100.0	106.56	100.0
Iso02550	longreads	99.9422	11	100.0	100.0	100.0	99.37	100.0	100.0	100.0	100.0	100.0	106.56	100.0
Iso02550	shortreads	99.9781	11	100.0	100.0	100.0	99.37	100.0	100.0	100.0	100.0	100.0	106.56	100.0
Iso02551	hybrid	99.9733	7	.	.	100.0	99.37	100.0	100.0	100.0	100.0	.	106.56	.
Iso02551	longreads	99.9410	6	.	.	100.0	.	100.0	100.0	100.0	100.0	.	106.56	.
Iso02551	shortreads	99.9775	7	.	.	100.0	99.37	100.0	100.0	100.0	100.0	.	106.56	.
Iso02552	hybrid	99.9702	11	100.0	100.0	100.0	99.37	100.0	100.0	100.0	100.0	100.0	106.56	100.0
Iso02552	longreads	99.9340	11	100.0	100.0	100.0	99.37	100.0	100.0	100.0	100.0	100.0	106.56	100.0
Iso02552	shortreads	99.9792	11	100.0	100.0	100.0	99.37	100.0	100.0	100.0	100.0	100.0	106.56	100.0
Iso02553	hybrid	99.9731	11	100.0	100.0	100.0	99.37	100.0	100.0	100.0	100.0	100.0	106.56	100.0
Iso02553	longreads	99.9410	11	100.0	100.0	100.0	99.37	100.0	100.0	100.0	100.0	100.0	106.56	100.0
Iso02553	shortreads	99.9797	11	100.0	100.0	100.0	99.37	100.0	100.0	100.0	100.0	100.0	106.56	100.0
Iso02554	hybrid	99.9739	11	100.0	100.0	100.0	99.37	100.0	100.0	100.0	100.0	100.0	106.56	100.0
Iso02554	longreads	99.9408	11	100.0	100.0	100.0	99.37	100.0	100.0	100.0	100.0	100.0	106.56	100.0
Iso02554	shortreads	99.9787	11	100.0	100.0	100.0	99.37	100.0	100.0	100.0	100.0	100.0	106.56	100.0
Iso02555	hybrid	99.9753	11	100.0	100.0	100.0	99.37	100.0	100.0	100.0	100.0	100.0	106.56	100.0
Iso02555	longreads	99.9405	10	100.0	100.0	100.0	.	100.0	100.0	100.0	100.0	100.0	106.56	100.0
Iso02555	shortreads	99.9765	11	100.0	100.0	100.0	99.37	100.0	100.0	100.0	100.0	100.0	106.56	100.0
Iso02556	hybrid	99.9735	11	100.0	100.0	100.0	99.37	100.0	100.0	100.0	100.0	100.0	106.56	100.0
Iso02556	longreads	99.9401	11	100.0	100.0	100.0	99.37	100.0	100.0	100.0	100.0	100.0	106.56	100.0
Iso02556	shortreads	99.9781	11	100.0	100.0	100.0	99.37	100.0	100.0	100.0	100.0	100.0	106.56	100.0
Iso02557	hybrid	99.9729	11	100.0	100.0	100.0	99.37	100.0	100.0	100.0	100.0	100.0	106.56	100.0
Iso02557	longreads	99.9430	10	100.0	100.0	100.0	.	100.0	100.0	100.0	100.0	100.0	106.56	100.0
Iso02557	shortreads	99.9804	11	100.0	100.0	100.0	99.37	100.0	100.0	100.0	100.0	100.0	106.56	100.0
Iso02558	hybrid	99.9724	11	100.0	100.0	100.0	99.37	100.0	100.0	100.0	100.0	100.0	106.56	100.0
Iso02558	longreads	99.9435	10	100.0	100.0	100.0	.	100.0	100.0	100.0	100.0	100.0	106.56	100.0
Iso02558	shortreads	99.9773	11	100.0	100.0	100.0	99.37	100.0	100.0	100.0	100.0	100.0	106.56	100.0
Iso02559	hybrid	99.9790	11	100.0	100.0	100.0	99.37	100.0	100.0	100.0	100.0	100.0	106.56	100.0
Iso02559	longreads	99.9468	10	100.0	100.0	100.0	.	100.0	100.0	100.0	100.0	100.0	106.56	100.0
Iso02559	shortreads	99.9833	11	100.0	100.0	100.0	99.37	100.0	100.0	100.0	100.0	100.0	106.56	100.0
Iso02560	hybrid	99.9743	11	100.0	100.0	100.0	99.37	100.0	100.0	100.0	100.0	100.0	106.56	100.0
Iso02560	longreads	99.9409	10	100.0	100.0	100.0	.	100.0	100.0	100.0	100.0	100.0	106.56	100.0
Iso02560	shortreads	99.9746	11	100.0	100.0	100.0	99.37	100.0	100.0	100.0	100.0	100.0	106.56	100.0
Iso02561	hybrid	99.9820	11	100.0	100.0	100.0	99.37	100.0	100.0	100.0	100.0	100.0	106.56	100.0
Iso02561	longreads	99.9394	10	100.0	100.0	100.0	.	100.0	100.0	100.0	100.0	100.0	106.56	100.0
Iso02561	shortreads	99.9775	11	100.0	100.0	100.0	99.37	100.0	100.0	100.0	100.0	100.0	106.56	100.0
Iso02563	hybrid	99.9768	11	100.0	100.0	100.0	99.37	100.0	100.0	100.0	100.0	100.0	106.56	100.0
Iso02563	longreads	99.9461	10	100.0	100.0	100.0	.	100.0	100.0	100.0	100.0	100.0	106.56	100.0
Iso02563	shortreads	99.9793	11	100.0	100.0	100.0	99.37	100.0	100.0	100.0	100.0	100.0	106.56	100.0
Iso02564	hybrid	99.9714	11	100.0	100.0	100.0	99.37	100.0	100.0	100.0	100.0	100.0	106.56	100.0
Iso02564	longreads	99.9360	10	100.0	100.0	100.0	.	100.0	100.0	100.0	100.0	100.0	106.56	100.0
Iso02564	shortreads	99.9774	11	100.0	100.0	100.0	99.37	100.0	100.0	100.0	100.0	100.0	106.56	100.0
Iso02565	hybrid	99.9736	11	100.0	100.0	100.0	99.37	100.0	100.0	100.0	100.0	100.0	106.56	100.0
Iso02565	longreads	99.9336	10	100.0	100.0	100.0	.	100.0	100.0	100.0	100.0	100.0	106.56	100.0
Iso02565	shortreads	99.9782	11	100.0	100.0	100.0	99.37	100.0	100.0	100.0	100.0	100.0	106.56	100.0
Iso02566	hybrid	99.9734	11	100.0	100.0	100.0	99.37	100.0	100.0	100.0	100.0	100.0	106.56	100.0
Iso02566	longreads	99.9401	10	100.0	100.0	100.0	.	100.0	100.0	100.0	100.0	100.0	106.56	100.0
Iso02566	shortreads	99.9775	11	100.0	100.0	100.0	99.37	100.0	100.0	100.0	100.0	100.0	106.56	100.0
Iso02567	hybrid	99.9758	11	100.0	100.0	100.0	99.37	100.0	100.0	100.0	100.0	100.0	106.56	100.0
Iso02567	longreads	99.9457	11	100.0	100.0	100.0	99.37	100.0	100.0	100.0	100.0	100.0	106.56	100.0
Iso02567	shortreads	99.9784	11	100.0	100.0	100.0	99.37	100.0	100.0	100.0	100.0	100.0	106.56	100.0
Iso02568	hybrid	99.9769	7	.	.	100.0	99.37	100.0	100.0	100.0	100.0	.	106.56	.
Iso02568	longreads	99.9490	6	.	.	100.0	.	100.0	100.0	100.0	100.0	.	106.56	.
Iso02568	shortreads	99.9743	7	.	.	100.0	99.37	100.0	100.0	100.0	100.0	.	106.56	.
Iso02569	hybrid	99.9756	11	100.0	100.0	100.0	99.37	100.0	100.0	100.0	100.0	100.0	106.56	100.0
Iso02569	longreads	99.9409	10	100.0	100.0	100.0	.	100.0	100.0	100.0	100.0	100.0	106.56	100.0
Iso02569	shortreads	99.9774	11	100.0	100.0	100.0	99.37	100.0	100.0	100.0	100.0	100.0	106.56	100.0
Iso02570	hybrid	99.9734	11	100.0	100.0	100.0	99.37	100.0	100.0	100.0	100.0	100.0	106.56	100.0
Iso02570	longreads	99.9367	11	100.0	100.0	100.0	99.37	100.0	100.0	100.0	100.0	100.0	106.56	100.0
Iso02570	shortreads	99.9791	11	100.0	100.0	100.0	99.37	100.0	100.0	100.0	100.0	100.0	106.56	100.0
Iso02571	hybrid	99.9747	11	100.0	100.0	100.0	99.37	100.0	100.0	100.0	100.0	100.0	106.56	100.0
Iso02571	longreads	99.9364	10	100.0	100.0	100.0	.	100.0	100.0	100.0	100.0	100.0	106.56	100.0
Iso02571	shortreads	99.9792	11	100.0	100.0	100.0	99.37	100.0	100.0	100.0	100.0	100.0	106.56	100.0
Iso02572	hybrid	99.9756	11	100.0	100.0	100.0	99.37	100.0	100.0	100.0	100.0	100.0	106.56	100.0
Iso02572	longreads	99.9486	10	100.0	100.0	100.0	.	100.0	100.0	100.0	100.0	100.0	106.56	100.0
Iso02572	shortreads	99.9738	11	100.0	100.0	100.0	99.37	100.0	100.0	100.0	100.0	100.0	106.56	100.0
Iso02573	hybrid	99.9677	11	100.0	100.0	100.0	99.37	100.0	100.0	100.0	100.0	100.0	106.56	100.0
Iso02573	longreads	99.9385	11	100.0	100.0	100.0	99.37	100.0	100.0	100.0	100.0	100.0	106.56	100.0
Iso02573	shortreads	99.9743	11	100.0	100.0	100.0	99.37	100.0	100.0	100.0	100.0	100.0	106.56	100.0
Iso02575	hybrid	99.9771	7	.	.	100.0	99.37	100.0	100.0	100.0	100.0	.	106.56	.
Iso02575	longreads	99.9483	7	.	.	100.0	99.37	100.0	100.0	100.0	100.0	.	106.56	.
Iso02575	shortreads	99.9774	7	.	.	100.0	99.37	100.0	100.0	100.0	100.0	.	106.56	.
Iso02577	hybrid	99.9806	11	100.0	100.0	100.0	99.37	100.0	100.0	100.0	100.0	100.0	106.56	100.0
Iso02577	longreads	99.9478	10	100.0	100.0	100.0	.	100.0	100.0	100.0	100.0	100.0	106.56	100.0
Iso02577	shortreads	99.9762	11	100.0	100.0	100.0	99.37	100.0	100.0	100.0	100.0	100.0	106.56	100.0
Iso02579	hybrid	99.9747	11	100.0	100.0	100.0	99.37	100.0	100.0	100.0	100.0	100.0	106.56	100.0

Iso02579	longreads	99.9445	11	100.0	100.0	100.0	99.37	100.0	100.0	100.0	100.0	100.0	106.56	100.0
Iso02579	shortreads	99.9760	11	100.0	100.0	100.0	99.37	100.0	100.0	100.0	100.0	100.0	106.56	100.0
Iso02580	hybrid	99.9709	11	100.0	100.0	100.0	99.37	100.0	100.0	100.0	100.0	100.0	106.56	100.0
Iso02580	longreads	99.9411	10	100.0	100.0	100.0	.	100.0	100.0	100.0	100.0	100.0	106.56	100.0
Iso02580	shortreads	99.9752	11	100.0	100.0	100.0	99.37	100.0	100.0	100.0	100.0	100.0	106.56	100.0
Iso02581	hybrid	99.9792	11	100.0	100.0	100.0	99.37	100.0	100.0	100.0	100.0	100.0	106.56	100.0
Iso02581	longreads	99.9485	10	100.0	100.0	100.0	.	100.0	100.0	100.0	100.0	100.0	106.56	100.0
Iso02581	shortreads	99.9754	11	100.0	100.0	100.0	99.37	100.0	100.0	100.0	100.0	100.0	106.56	100.0
Iso02582	hybrid	99.9744	11	100.0	100.0	100.0	99.37	100.0	100.0	100.0	100.0	100.0	106.56	100.0
Iso02582	longreads	99.9417	10	100.0	100.0	100.0	.	100.0	100.0	100.0	100.0	100.0	106.56	100.0
Iso02582	shortreads	99.9834	11	100.0	100.0	100.0	99.37	100.0	100.0	100.0	100.0	100.0	106.56	100.0
Iso02583	hybrid	97.9259	4	.	.	100.0	99.37	.	100.0	.	.	.	106.56	.
Iso02583	longreads	97.9168	4	.	.	100.0	99.37	.	100.0	.	.	.	106.56	.
Iso02583	shortreads	98.0757	4	.	.	100.0	99.37	.	100.0	.	.	.	106.56	.
Iso02584	hybrid	99.9771	11	100.0	100.0	100.0	99.37	100.0	100.0	100.0	100.0	100.0	106.56	100.0
Iso02584	longreads	99.9458	10	100.0	100.0	100.0	.	100.0	100.0	100.0	100.0	100.0	106.56	100.0
Iso02584	shortreads	99.9778	11	100.0	100.0	100.0	99.37	100.0	100.0	100.0	100.0	100.0	106.56	100.0
Iso02585	hybrid	99.9731	11	100.0	100.0	100.0	99.37	100.0	100.0	100.0	100.0	100.0	106.56	100.0
Iso02585	longreads	99.9467	11	100.0	100.0	100.0	99.37	100.0	100.0	100.0	100.0	100.0	106.56	100.0
Iso02585	shortreads	99.9791	11	100.0	100.0	100.0	99.37	100.0	100.0	100.0	100.0	100.0	106.56	100.0
Iso02586	hybrid	99.9733	11	100.0	100.0	100.0	99.37	100.0	100.0	100.0	100.0	100.0	106.56	100.0
Iso02586	longreads	99.9437	10	100.0	100.0	100.0	.	100.0	100.0	100.0	100.0	100.0	106.56	100.0
Iso02586	shortreads	99.9829	11	100.0	100.0	100.0	99.37	100.0	100.0	100.0	100.0	100.0	106.56	100.0
Iso02587	hybrid	99.9723	11	100.0	100.0	100.0	99.37	100.0	100.0	100.0	100.0	100.0	106.56	100.0
Iso02587	longreads	99.9438	11	100.0	100.0	100.0	99.37	100.0	100.0	100.0	100.0	100.0	106.56	100.0
Iso02587	shortreads	99.9829	11	100.0	100.0	100.0	99.37	100.0	100.0	100.0	100.0	100.0	106.56	100.0
Iso02588	hybrid	99.9749	11	100.0	100.0	100.0	99.37	100.0	100.0	100.0	100.0	100.0	106.56	100.0
Iso02588	longreads	99.9528	10	100.0	100.0	100.0	.	100.0	100.0	100.0	100.0	100.0	106.56	100.0
Iso02588	shortreads	99.9789	11	100.0	100.0	100.0	99.37	100.0	100.0	100.0	100.0	100.0	106.56	100.0
Iso02589	hybrid	99.9738	11	100.0	100.0	100.0	99.37	100.0	100.0	100.0	100.0	100.0	106.56	100.0
Iso02589	longreads	99.9443	10	100.0	100.0	100.0	.	100.0	100.0	100.0	100.0	100.0	106.56	100.0
Iso02589	shortreads	99.9770	11	100.0	100.0	100.0	99.37	100.0	100.0	100.0	100.0	100.0	106.56	100.0
Iso02590	hybrid	99.9750	11	100.0	100.0	100.0	99.37	100.0	100.0	100.0	100.0	100.0	106.56	100.0
Iso02590	longreads	99.9486	10	100.0	100.0	100.0	.	100.0	100.0	100.0	100.0	100.0	106.56	100.0
Iso02590	shortreads	99.9810	11	100.0	100.0	100.0	99.37	100.0	100.0	100.0	100.0	100.0	106.56	100.0
Iso02591	hybrid	99.9775	11	100.0	100.0	100.0	99.37	100.0	100.0	100.0	100.0	100.0	106.56	100.0
Iso02591	longreads	99.9456	11	100.0	100.0	100.0	99.37	100.0	100.0	100.0	100.0	100.0	106.56	100.0
Iso02591	shortreads	99.9805	11	100.0	100.0	100.0	99.37	100.0	100.0	100.0	100.0	100.0	106.56	100.0
Iso02592	hybrid	99.9678	11	100.0	100.0	100.0	99.37	100.0	100.0	100.0	100.0	100.0	106.56	100.0
Iso02592	longreads	99.9428	10	100.0	100.0	100.0	.	100.0	100.0	100.0	100.0	100.0	106.56	100.0
Iso02592	shortreads	99.9776	11	100.0	100.0	100.0	99.37	100.0	100.0	100.0	100.0	100.0	106.56	100.0
Iso02593	hybrid	99.9774	11	100.0	100.0	100.0	99.37	100.0	100.0	100.0	100.0	100.0	106.56	100.0
Iso02593	longreads	99.9447	10	100.0	100.0	100.0	.	100.0	100.0	100.0	100.0	100.0	106.56	100.0
Iso02593	shortreads	99.9818	11	100.0	100.0	100.0	99.37	100.0	100.0	100.0	100.0	100.0	106.56	100.0
Iso02594	hybrid	99.9766	11	100.0	100.0	100.0	99.37	100.0	100.0	100.0	100.0	100.0	106.56	100.0
Iso02594	longreads	99.9458	10	100.0	100.0	100.0	.	100.0	100.0	100.0	100.0	100.0	106.56	100.0
Iso02594	shortreads	99.9796	11	100.0	100.0	100.0	99.37	100.0	100.0	100.0	100.0	100.0	106.56	100.0
Iso02595	hybrid	99.9748	11	100.0	100.0	100.0	99.37	100.0	100.0	100.0	100.0	100.0	106.56	100.0
Iso02595	longreads	99.9519	10	100.0	100.0	100.0	.	100.0	100.0	100.0	100.0	100.0	106.56	100.0
Iso02595	shortreads	99.9775	11	100.0	100.0	100.0	99.37	100.0	100.0	100.0	100.0	100.0	106.56	100.0
Iso02597	hybrid	99.9790	11	100.0;<br>100.0;<br>100.0	100.0;<br>100.0;<br>100.0	100.0	99.37	100.0	100.0	100.0	100.0	100.0;<br>100.0;<br>100.0	106.56	100.0;<br>100.0;<br>100.0
Iso02597	longreads	99.9476	11	100.0;<br>100.0;<br>100.0	100.0;<br>100.0;<br>100.0	100.0	99.37	100.0	100.0	100.0	100.0	100.0;<br>100.0;<br>100.0	106.56	100.0;<br>100.0;<br>100.0
Iso02597	shortreads	99.9810	11	100.0	100.0	100.0	99.37	100.0	100.0	100.0	100.0	100.0	106.56	100.0
