# Supplementary material for: Prediction of Antibiotic Susceptibility Profiles of Vibrio cholerae Isolates From Whole Genome Illumina and Nanopore Sequencing Data: CholerAegon
Source: Front Microbiol. 2022 Jun 22;13:909692. doi: 10.3389/fmicb.2022.909692 (PMC9257098; doi:10.3389/fmicb.2022.909692)
Supplement: Supplementary file 3 [file Table_3.pdf]

Table 3: AMR genes found in temporal subsets of isolate Iso02507. Sequencing data was separated in cumulative subsets defined by sequencing start time of the nanopore reads. These subsets were assembled with scanned for AMR genes with CholerAegon. parE – Escherichia coli parE conferring resistance to fluoroquinolones.

	Assembly number	Sample	Assembly method	FastANI %	# Genes found	catB9	APH(3”)-Ib	APH(6)-Id	CRP	Vibrio cholerae varG	almG	dfrA1	floR	rsmA	sul2	parE
0	1	Iso02507_0004min	longreads	98.9654	0	.	.	.	.	.	.	.	.	.	.	.
1	2	Iso02507_0005min	longreads	98.9755	0	.	.	.	.	.	.	.	.	.	.	.
2	3	Iso02507_0006min	longreads	99.3675	0	.	.	.	.	.	.	.	.	.	.	.
3	4	Iso02507_0007min	longreads	99.0984	0	.	.	.	.	.	.	.	.	.	.	.
4	5	Iso02507_0008min	longreads	99.5066	0	.	.	.	.	.	.	.	.	.	.	.
5	6	Iso02507_0009min	longreads	99.2482	0	.	.	.	.	.	.	.	.	.	.	.
6	7	Iso02507_0010min	longreads	100.0000	0	.	.	.	.	.	.	.	.	.	.	.
7	8	Iso02507_0011min	longreads	99.4252	0	.	.	.	.	.	.	.	.	.	.	.
8	9	Iso02507_0012min	longreads	99.1757	0	.	.	.	.	.	.	.	.	.	.	.
9	10	Iso02507_0013min	longreads	99.1241	0	.	.	.	.	.	.	.	.	.	.	.
10	11	Iso02507_0014min	longreads	99.1166	0	.	.	.	.	.	.	.	.	.	.	.
11	12	Iso02507_0015min	longreads	99.1376	1	99.37	.	.	.	.	.	.	.	.	.	.
12	13	Iso02507_0016min	longreads	99.2256	1	99.37	.	.	.	.	.	.	.	.	.	.
13	14	Iso02507_0017min	longreads	99.1334	1	99.37	.	.	.	.	.	.	.	.	.	.
14	15	Iso02507_0018min	longreads	99.1229	1	99.37	.	.	.	.	.	.	.	.	.	.
15	16	Iso02507_0019min	longreads	99.0979	1	99.37	.	.	.	.	.	.	.	.	.	.
16	17	Iso02507_0020min	longreads	99.1487	1	99.37	.	.	.	.	.	.	.	.	.	.
17	18	Iso02507_0025min	longreads	99.2112	4	100.0	.	.	100.0	.	.	100.0	.	106.56	.	.
18	19	Iso02507_0030min	longreads	99.3945	8	100.0	100.0	100.0	100.0	.	.	100.0	99.84	106.56	99.88	.
19	20	Iso02507_0035min	longreads	99.5359	8	100.0	99.88	100.0	100.0	.	.	100.0	99.84	106.56	99.88	.
20	21	Iso02507_0040min	longreads	99.6045	10	100.0	100.0	100.0	100.0	100.0;99.32	99.76	100.0	99.84	106.56	99.88	.
21	22	Iso02507_0045min	longreads	99.6927	10	100.0	100.0	100.0	100.0	99.91;99.83	99.88;99.88	100.0	100.0	106.56	99.88	.
22	23	Iso02507_0050min	longreads	99.7700	8	100.0	100.0	100.0	100.0	.	.	100.0	100.0	106.56	99.88	.
23	24	Iso02507_0060min	longreads	99.8376	10	100.0	100.0	100.0	100.0	100.0	100.0	100.0	100.0	106.56	99.88	.
24	25	Iso02507_0070min	longreads	99.8817	10	100.0	100.0	99.88	100.0	100.0	100.0	100.0	100.0	106.56	99.88	.
25	26	Iso02507_0080min	longreads	99.8959	10	100.0	100.0	100.0	100.0	100.0	100.0	100.0	100.25	106.56	100.0	.
26	27	Iso02507_0090min	longreads	99.9054	10	100.0	100.0	115.11	100.0	100.0;99.57	100.0;99.15	100.0	100.0	106.56	99.88	.
27	28	Iso02507_0100min	longreads	99.9049	10	100.0	100.0	115.11	100.0	100.0	100.0	100.0	100.0	106.56	99.88	.
28	29	Iso02507_0110min	longreads	99.9155	10	100.0	100.0	115.11	100.0	100.0	100.0	100.0	100.0	106.56	100.0	.
29	30	Iso02507_0120min	longreads	99.9141	10	100.0	100.0	100.0	100.0	100.0	100.0	100.0	100.0	106.56	100.0	.
30	31	Iso02507_0130min	longreads	99.9173	10	100.0	100.0	100.0	100.0	100.0	100.0	100.0	100.0	106.56	100.0	.
31	32	Iso02507_0140min	longreads	99.9248	10	100.0	100.0	100.0	100.0	100.0	100.0	100.0	100.0	106.56	100.0	.
32	33	Iso02507_0150min	longreads	99.9199	10	100.0	100.0	100.0	100.0	100.0	100.0	100.0	100.0	106.56	100.0	.
33	34	Iso02507_0160min	longreads	99.9211	10	100.0	100.0	100.0	100.0	100.0	100.0	100.0	100.0	106.56	99.88	.
34	35	Iso02507_0170min	longreads	99.9208	10	100.0	100.0	100.0	100.0	100.0	100.0	100.0	100.0	106.56	99.88	.
35	36	Iso02507_0180min	longreads	99.9270	10	100.0	100.0	100.0	100.0	100.0	100.0	100.0	100.0	106.56	99.88	.
36	37	Iso02507_0190min	longreads	99.9230	10	100.0	100.0	100.0	100.0	100.0	100.0	100.0	100.0	106.56	99.88	.
37	38	Iso02507_0200min	longreads	99.9225	10	100.0	100.0	100.0	100.0	100.0	100.0	100.0	100.0	106.56	99.88	.
38	39	Iso02507_0250min	longreads	99.9214	10	100.0	100.0	100.0	100.0	100.0	100.0	100.0	100.0	106.56	99.88	.
39	40	Iso02507_0300min	longreads	99.9309	10	100.0	100.0	100.0	100.0	100.0	100.0	100.0	100.0	106.56	99.88	.
40	41	Iso02507_0350min	longreads	99.9287	10	100.0	100.0	100.0	100.0	100.0	100.0	100.0	100.0	106.56	100.0	.
41	42	Iso02507_0400min	longreads	99.9241	10	100.0	100.0	100.0	100.0	100.0	100.0	100.0	100.0	106.56	100.0	.
42	43	Iso02507_0450min	longreads	99.9352	10	100.0	100.0	100.0	100.0	100.0	100.0	100.0	100.0	106.56	100.0	.
43	44	Iso02507_0500min	longreads	99.9329	10	100.0	100.0	100.0	100.0	100.0	100.0	100.0	100.0	106.56	100.0	.
44	45	Iso02507_0550min	longreads	99.9352	10	100.0	100.0	100.0	100.0	100.0	100.0	100.0	100.0	106.56	100.0	.
45	46	Iso02507_0600min	longreads	99.9442	11	100.0	100.0	100.0	100.0	100.0	100.0	100.0	100.0	106.56	100.0	99.37
46	47	Iso02507_0650min	longreads	99.9355	10	100.0	100.0	100.0	100.0	100.0	100.0	100.0	100.0	106.56	100.0	.
47	48	Iso02507_0700min	longreads	99.9404	10	100.0	100.0	100.0	100.0	100.0	100.0	100.0	100.0	106.56	100.0	.
48	49	Iso02507_0750min	longreads	99.9409	10	100.0	100.0	100.0	100.0	100.0	100.0	100.0	100.0	106.56	100.0	.
49	50	Iso02507_0800min	longreads	99.9418	10	100.0	100.0	100.0	100.0	100.0	100.0	100.0	100.0	106.56	100.0	.
50	51	Iso02507_0850min	longreads	99.9425	10	100.0	100.0	100.0	100.0	100.0	100.0	100.0	100.0	106.56	100.0	.
51	52	Iso02507_0900min	longreads	99.9395	10	100.0	100.0	100.0	100.0	100.0	100.0	100.0	100.0	106.56	100.0	.
52	53	Iso02507_0950min	longreads	99.9373	10	100.0	100.0	100.0	100.0	100.0	100.0	100.0	100.0	106.56	100.0	.
53	54	Iso02507_1000min	longreads	99.9399	11	100.0	100.0	100.0	100.0	100.0	100.0	100.0	100.0	106.56	100.0	99.37
54	55	Iso02507_1100min	longreads	99.9403	10	100.0	100.0	100.0	100.0	100.0	100.0	100.0	100.0	106.56	100.0	.
55	56	Iso02507_1200min	longreads	99.9386	10	100.0	100.0	100.0	100.0	100.0	100.0	100.0	100.0	106.56	100.0	.
56	57	Iso02507_1300min	longreads	99.9390	10	100.0	100.0	100.0	100.0	100.0	100.0	100.0	100.0	106.56	100.0	.
57	58	Iso02507_1400min	longreads	99.9420	10	100.0	100.0	100.0	100.0	100.0	100.0	100.0	100.0	106.56	100.0	.
58	59	Iso02507_1500min	longreads	99.9452	10	100.0	100.0	100.0	100.0	100.0	100.0	100.0	100.0	106.56	100.0	.
59	60	Iso02507_1600min	longreads	99.9450	10	100.0	100.0	100.0	100.0	100.0	100.0	100.0	100.0	106.56	100.0	.

60	61	Iso02507_1700min	longreads	99.9441	10	100.0	100.0	100.0	100.0	100.0	100.0	100.0	100.0	106.56	100.0	.
61	62	Iso02507_1800min	longreads	99.9408	11	100.0	100.0	100.0	100.0	100.0	100.0	100.0	100.0	106.56	100.0	99.37
62	63	Iso02507_1900min	longreads	99.9398	10	100.0	100.0	100.0	100.0	100.0	100.0	100.0	100.0	106.56	100.0	.
63	64	Iso02507_2000min	longreads	99.9414	10	100.0	100.0	100.0	100.0	100.0	100.0	100.0	100.0	106.56	100.0	.
64	65	Iso02507_2100min	longreads	99.9410	10	100.0	100.0	100.0	100.0	100.0	100.0	100.0	100.0	106.56	100.0	.
65	66	Iso02507_2200min	longreads	99.9426	10	100.0	100.0	100.0	100.0	100.0	100.0	100.0	100.0	106.56	100.0	.
66	67	Iso02507_2300min	longreads	99.9415	10	100.0	100.0	100.0	100.0	100.0	100.0	100.0	100.0	106.56	100.0	.
67	68	Iso02507_2400min	longreads	99.9467	10	100.0	100.0	100.0	100.0	100.0	100.0	100.0	100.0	106.56	100.0	.
68	69	Iso02507_2500min	longreads	99.9408	10	100.0	100.0	100.0	100.0	100.0	100.0	100.0	100.0	106.56	100.0	.
69	70	Iso02507_2600min	longreads	99.9469	10	100.0	100.0	100.0	100.0	100.0	100.0	100.0	100.0	106.56	100.0	.
70	71	Iso02507_2700min	longreads	99.9405	10	100.0	100.0	100.0	100.0	100.0	100.0	100.0	100.0	106.56	100.0	.
71	72	Iso02507_2800min	longreads	99.9393	10	100.0	100.0	100.0	100.0	100.0	100.0	100.0	100.0	106.56	100.0	.
