# Supplementary material for: Prediction of Antibiotic Susceptibility Profiles of Vibrio cholerae Isolates From Whole Genome Illumina and Nanopore Sequencing Data: CholerAegon
Source: Front Microbiol. 2022 Jun 22;13:909692. doi: 10.3389/fmicb.2022.909692 (PMC9257098; doi:10.3389/fmicb.2022.909692)
Supplement: Supplementary file 4 [file Table_4.pdf]

Table 4: Comparison of *in-silico* prediction of antimicrobial resistance from sequencing data against antimicrobial susceptibility testing (AST) results. We used CholerAegon to assemble 82 isolates and subsequently predict resistances. For 80 of these isolates, AST results are available, which assigned a phenotype of "susceptible" (S), intermediate" (I), "susceptible dose-dependent" (SD) or "resistant" (R) with regard to seven tested antibiotics. We consider a prediction correct if for the "susceptible" (S) phenotype, no resistance has been predicted (no relevant gene or gene variant present), or if for the "intermediate" (I), "susceptible dose-dependent" (SD) and "resistant" (R) statuses, the resistance was predicted (relevant gene or gene variant found). All other combinations are considered false predictions. The result 'S_yes' is marked in correct to note that while the prediction is wrong, the presence of the gene(s) does directly confer a resistant phenotype. S – susceptible; SD – susceptible dose-dependent; R – resistant; I – intermediate (according to CLSI guidelines 2015); AMP – ampicillin; C – chloramphenicol; CIP – ciprofloxacin; CN – gentamicin A; TE – tetracycline; SxT – trimethoprim-sulfamethoxazole; NA – nalidixic acid; EC parE – Escherichia coli parE conferring resistance to fluoroquinolones.

Antibiotic	AMP	C	CIP	CN	TE	SxT	NA
Genes	-	catB9, floR	EC parE	-	-	dfrA1 + sul2	EC parE
Prediction %	25% 75%	4% 96%	78% 22%	100% 0%	100% 0%	94% 6%	79% 21%
Correct	20	3	62	80	80	75	63
False	60	77	18	0	0	5	17
Iso02501	S_no	S_yes	S_yes	S_no	S_no	R_yes	S_yes
Iso02502	S_no	S_yes	S_yes	S_no	S_no	R_yes	S_yes
Iso02503	S_no	S_yes	S_yes	S_no	S_no	R_yes	S_yes
Iso02504	S_no	S_yes	S_yes	S_no	S_no	R_yes	S_yes
Iso02506	S_no	S_yes	S_yes	S_no	S_no	R_no	S_yes
Iso02507	S_no	S_yes	S_yes	S_no	S_no	R_yes	S_yes
Iso02508	S_no	S_yes	S_yes	S_no	S_no	R_yes	S_yes
Iso02509	S_no	S_yes	S_yes	S_no	S_no	R_yes	S_yes
Iso02510	S_no	S_yes	S_yes	S_no	S_no	R_yes	S_yes
Iso02511	S_no	S_yes	S_yes	S_no	S_no	R_yes	S_yes
Iso02512	S_no	S_yes	S_yes	S_no	S_no	R_yes	S_yes
Iso02513	S_no	S_yes	S_yes	S_no	S_no	R_yes	S_yes
Iso02514	S_no	S_yes	S_yes	S_no	S_no	R_yes	S_yes
Iso02515	S_no	S_yes	S_yes	S_no	S_no	R_yes	S_yes
Iso02517	S_no	S_yes	S_yes	S_no	S_no	R_yes	S_yes
Iso02518	S_no	S_yes	S_yes	S_no	S_no	R_yes	S_yes
Iso02519	I_no	S_yes	SD_yes	S_no	S_no	S_no	R_yes
Iso02520	S_no	S_yes	S_yes	S_no	S_no	R_yes	S_yes
Iso02522	I_no	S_yes	SD_yes	S_no	S_no	R_yes	R_yes
Iso02523	I_no	S_yes	SD_yes	S_no	S_no	R_yes	R_yes
Iso02524	I_no	S_yes	SD_yes	S_no	S_no	R_yes	R_yes
Iso02525	I_no	S_yes	SD_yes	S_no	S_no	R_yes	R_yes
Iso02527	I_no	S_yes	SD_yes	S_no	S_no	R_yes	R_yes
Iso02529	I_no	S_yes	SD_yes	S_no	S_no	R_yes	R_yes
Iso02532	I_no	S_yes	SD_yes	S_no	S_no	R_yes	R_yes
Iso02533	I_no	S_yes	SD_yes	S_no	S_no	R_yes	R_yes
Iso02534	I_no	S_yes	SD_yes	S_no	S_no	R_yes	R_yes
Iso02535	I_no	S_yes	SD_yes	S_no	S_no	R_yes	R_yes
Iso02536	I_no	S_yes	SD_yes	S_no	S_no	R_yes	R_yes
Iso02537	I_no	S_yes	SD_yes	S_no	S_no	R_yes	R_yes
Iso02538	S_no	S_no	SD_yes	S_no	S_no	S_no	R_yes
Iso02539	S_no	S_no	S_yes	S_no	S_no	S_no	R_yes
Iso02541	I_no	S_yes	SD_yes	S_no	S_no	R_yes	R_yes
Iso02542	I_no	S_yes	SD_yes	S_no	S_no	R_yes	R_yes
Iso02544	I_no	S_yes	SD_yes	S_no	S_no	R_yes	R_yes
Iso02545	I_no	S_yes	SD_yes	S_no	S_no	R_yes	R_yes
Iso02546	I_no	S_yes	SD_yes	S_no	S_no	R_yes	R_yes
Iso02547	I_no	S_yes	SD_yes	S_no	S_no	R_yes	R_yes
Iso02549	I_no	S_yes	SD_yes	S_no	S_no	R_yes	R_yes
Iso02550	I_no	S_yes	SD_yes	S_no	S_no	R_yes	R_yes
Iso02551	I_no	S_yes	SD_yes	S_no	S_no	R_no	R_yes
Iso02552	I_no	S_yes	SD_yes	S_no	S_no	R_yes	R_yes
Iso02553	I_no	S_yes	SD_yes	S_no	S_no	R_yes	R_yes
Iso02554	I_no	S_yes	SD_yes	S_no	S_no	R_yes	R_yes
Iso02555	I_no	S_yes	SD_yes	S_no	S_no	R_yes	R_yes
Iso02556	I_no	S_yes	SD_yes	S_no	S_no	R_yes	R_yes
Iso02557	I_no	S_yes	SD_yes	S_no	S_no	R_yes	R_yes
Iso02558	I_no	S_yes	SD_yes	S_no	S_no	R_yes	R_yes
Iso02559	I_no	S_yes	SD_yes	S_no	S_no	R_yes	R_yes
Iso02560	I_no	S_yes	SD_yes	S_no	S_no	R_yes	R_yes
Iso02561	I_no	S_yes	SD_yes	S_no	S_no	R_yes	R_yes
Iso02563	I_no	S_yes	SD_yes	S_no	S_no	R_yes	R_yes
Iso02564	I_no	S_yes	SD_yes	S_no	S_no	R_yes	R_yes
Iso02565	I_no	S_yes	SD_yes	S_no	S_no	R_yes	R_yes
Iso02566	I_no	S_yes	SD_yes	S_no	S_no	R_yes	R_yes
Iso02567	I_no	S_yes	SD_yes	S_no	S_no	R_yes	R_yes
Iso02568	I_no	S_yes	SD_yes	S_no	S_no	R_no	R_yes
Iso02569	I_no	S_yes	SD_yes	S_no	S_no	R_yes	R_yes
Iso02570	I_no	S_yes	SD_yes	S_no	S_no	R_yes	R_yes
Iso02571	I_no	S_yes	SD_yes	S_no	S_no	R_yes	R_yes
Iso02572	I_no	S_yes	SD_yes	S_no	S_no	R_yes	R_yes
Iso02573	I_no	S_yes	SD_yes	S_no	S_no	R_yes	R_yes
Iso02575	I_no	S_yes	SD_yes	S_no	S_no	R_no	R_yes
Iso02577	I_no	S_yes	SD_yes	S_no	S_no	R_yes	R_yes
Iso02579	I_no	S_yes	SD_yes	S_no	S_no	R_yes	R_yes
Iso02580	I_no	S_yes	SD_yes	S_no	S_no	R_yes	R_yes
Iso02581	I_no	S_yes	SD_yes	S_no	S_no	R_yes	R_yes
Iso02582	I_no	S_yes	SD_yes	S_no	S_no	R_yes	R_yes
Iso02583	S_no	S_no	SD_yes	S_no	S_no	R_no	R_yes
Iso02584	I_no	S_yes	SD_yes	S_no	S_no	R_yes	R_yes
Iso02585	I_no	S_yes	SD_yes	S_no	S_no	R_yes	R_yes
Iso02586	I_no	S_yes	SD_yes	S_no	S_no	R_yes	R_yes
Iso02587	I_no	S_yes	SD_yes	S_no	S_no	R_yes	R_yes
Iso02588	I_no	S_yes	SD_yes	S_no	S_no	R_yes	R_yes
Iso02589	I_no	S_yes	SD_yes	S_no	S_no	R_yes	R_yes
Iso02590	I_no	S_yes	SD_yes	S_no	S_no	R_yes	R_yes
Iso02591	I_no	S_yes	SD_yes	S_no	S_no	R_yes	R_yes
Iso02592	I_no	S_yes	SD_yes	S_no	S_no	R_yes	R_yes
Iso02593	I_no	S_yes	SD_yes	S_no	S_no	R_yes	R_yes
Iso02594	I_no	S_yes	SD_yes	S_no	S_no	R_yes	R_yes
Iso02595	nodata_no	nodata_yes	nodata_yes	nodata_no	nodata_no	nodata_yes	nodata_yes
Iso02597	nodata_no	nodata_yes	nodata_yes	nodata_no	nodata_no	nodata_yes	nodata_yes
