# Supplementary material for: Prediction of Antibiotic Susceptibility Profiles of Vibrio cholerae Isolates From Whole Genome Illumina and Nanopore Sequencing Data: CholerAegon
Source: Front Microbiol. 2022 Jun 22;13:909692. doi: 10.3389/fmicb.2022.909692 (PMC9257098; doi:10.3389/fmicb.2022.909692)
Supplement: Supplementary file 5 [file Table_5.pdf]

Table 5: Comparison of different tools for detection of antimicrobial resistance genes. We applied RGI[2], Abricate[3] (with the CARD database), AMRFinderPlus[4] and Resfinder[5] to the hybrid assemblies of 82 isolates of *V. cholerae*. RGI can detect the highest amount of genes and is uniquely able to use protein variant models to find gene variants. Abricate can detect gene loci at a higher completeness, being able to merge adjacent hits that were split in the underlying BLAST alignment. CholerAegon uses a combined approach, harnessing both RGI's and Abricate's advantages. RGI – Resistance Gene Identifier; COV – mean coverage of the reference gene sequence; EC parE – *Escherichia coli parE* conferring resistance to fluoroquinolones.

	RGI		Abricate		AMRFinderPlus		Resfinder	
AMR gene	# found	COV	# found	COV	# found	COV	# found	COV
APH(3”)-Ib	74	97.08	74	100.00	74	99.11	74	100.00
APH(6)-Id	74	100.00	74	100.00	74	100.00	74	100.00
CRP	82	100.00	82	99.68	0	–	0	–
Vibrio cholerae varG	79	95.90	79	100.00	79	100.00	0	–
almG	82	100.00	82	100.00	0	–	0	–
catB9	79	100.00	79	100.00	79	100.00	79	100.00
dfrA1	79	100.00	79	100.00	79	100.00	79	100.00
floR	74	99.94	74	100.00	74	99.96	74	99.92
rsmA	82	106.56	0	–	0	–	0	–
sul2	74	99.48	74	100.00	74	99.61	74	100.00
EC parE	82	99.37	0	–	0	–	0	–
