# Supplementary material for: Prediction of Antibiotic Susceptibility Profiles of Vibrio cholerae Isolates From Whole Genome Illumina and Nanopore Sequencing Data: CholerAegon
Source: Front Microbiol. 2022 Jun 22;13:909692. doi: 10.3389/fmicb.2022.909692 (PMC9257098; doi:10.3389/fmicb.2022.909692)
Supplement: Supplementary file 6 [file Image_1.pdf]

1 Supplementary Data

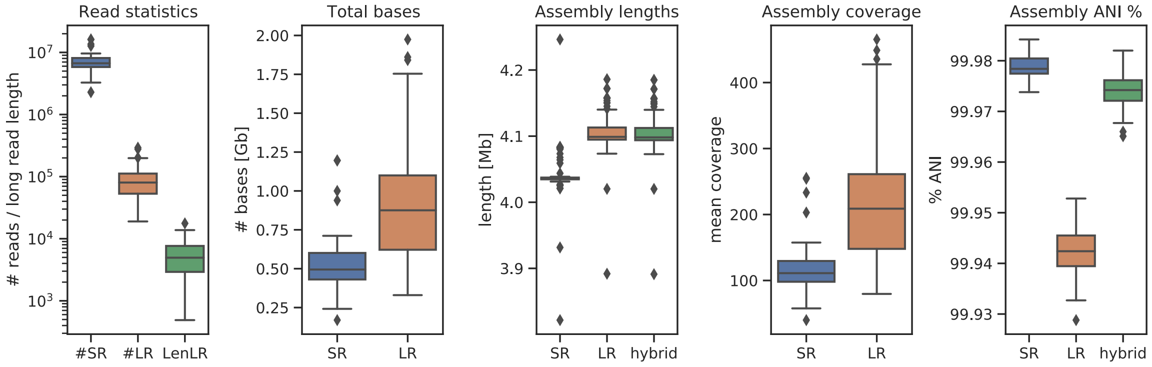

Figure 1: General statistics for sequencing data and assemblies. We sequenced 82 isolates of *V. cholerae* and performed individual assemblies with CholerAegon. ANI was determined against *V. cholerae* O1 biovar El Tor str. N16961, accession `NC_002505.1`. We excluded 3 isolates (Iso02538, Iso02539, Iso02583) from the ANI % boxplot, as these were found to represent a different serotype (NO O1 NO O139). SR – short reads; LR – long reads; LenLR – mean length of long reads; ANI – average nucleotide identity.
