# Supplementary material for: Prediction of Antibiotic Susceptibility Profiles of Vibrio cholerae Isolates From Whole Genome Illumina and Nanopore Sequencing Data: CholerAegon
Source: Front Microbiol. 2022 Jun 22;13:909692. doi: 10.3389/fmicb.2022.909692 (PMC9257098; doi:10.3389/fmicb.2022.909692)
Supplement: Supplementary file 7 [file Image_2.pdf]

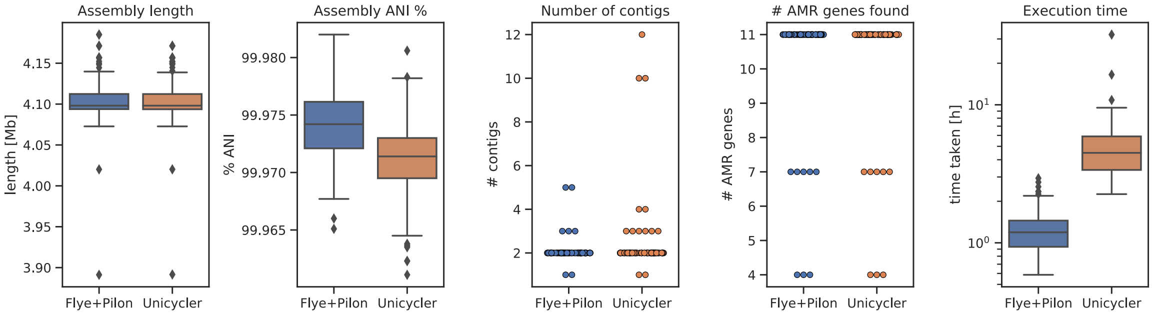

Figure 2: Comparison of hybrid assembly methods. We compared our long-reads-first assembly method (long read assembly with Flye followed by short read polishing with Pilon) to Unicycler [1], a short-reads-first hybrid assembler. The resulting assemblies for the 82 isolates of *V. cholerae* were almost identical in length. Average nucleotide identity was slightly lower for Unicycler. The results of AMR gene detection were exactly identical, but the Flye+Pilon approach is about 4 times faster than using Unicycler. Note that the shown execution time for the Flye+Pilon approach includes the execution time for bwa-mem2 and Medaka (in addition to Flye and Pilon themselves), which are not needed when using Unicycler. ANI was determined against *V. cholerae* O1 biovar El Tor str. N16961, accession `NC_002505.1`. We excluded 3 isolates (Iso02538, Iso02539, Iso02583) from the ANI % boxplot, as these were found to represent a different serotype (NO O1 NO O139). ANI – average nucleotide identity.
